# Facing lethal temperatures: Heat‐shock response in desert and temperate ants

**DOI:** 10.1002/ece3.10438

**Published:** 2023-09-14

**Authors:** Natalia de Souza Araujo, Rémy Perez, Quentin Willot, Matthieu Defrance, Serge Aron

**Affiliations:** ^1^ Department of Evolutionary Biology & Ecology Université Libre de Bruxelles Brussels Belgium; ^2^ Zoophysiology, Department of Biology Aarhus University Aarhus‐C Denmark; ^3^ Interuniversity Institute of Bioinformatics in Brussels Université Libre de Bruxelles Brussels Belgium

**Keywords:** heat stress, stress adaptation, thermal tolerance, transcriptomics

## Abstract

Global climate changes may cause profound effects on species adaptation, particularly in ectotherms for whom even moderate warmer temperatures can lead to disproportionate heat failure. Still, several organisms evolved to endure high desert temperatures. Here, we describe the thermal tolerance survival and the transcriptomic heat stress response of three genera of desert (*Cataglyphis*, *Melophorus*, and *Ocymyrmex*) and two of temperate ants (*Formica* and *Myrmica*) and explore convergent and specific adaptations. We found heat stress led to either a reactive or a constitutive response in desert ants: *Cataglyphis holgerseni* and *Melophorus bagoti* differentially regulated very few transcripts in response to heat (0.12% and 0.14%, respectively), while *Cataglyphis bombycina* and *Ocymyrmex robustior* responded with greater expression alterations (respectively affecting 0.6% and 1.53% of their transcriptomes). These two responsive mechanisms—reactive and constitutive—were related to individual thermal tolerance survival and convergently evolved in distinct desert ant genera. Moreover, in comparison with desert species, the two temperate ants differentially expressed thousands of transcripts more in response to heat stress (affecting 8% and 12.71% of *F*. *fusca* and *Myr*. *sabuleti* transcriptomes). In summary, we show that heat adaptation in thermophilic ants involved changes in the expression response. Overall, desert ants show reduced transcriptional alterations even when under high thermal stress, and their expression response may be either constitutive or reactive to temperature increase.

## Graphical abstract

Heat adaptation in thermophilic ants involved changes in their expression response pattern. Compared with desert species, temperate ants differentially expressed more genes in response to heat stress. Desert ants exhibited either a constitutive or a reactive response to temperature increase, and these responses were associated, respectively, with reduced or larger variation in individual survival during heat stress assays.

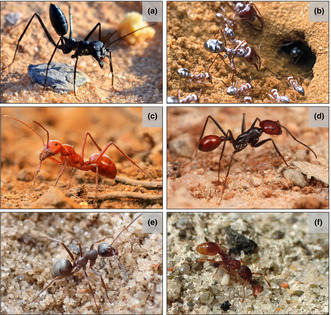

## 1 INTRODUCTION

Global climate changes affect species distribution, adaptation, and survival (Sala et al., 2000). Ectotherm species are particularly highly sensitive to temperature increases, and even moderately warmer temperatures may lead to a disproportionately increased heat failure (Jørgensen et al., 2022). To establish and improve predictive models and management plans to control biodiversity loss, it is, therefore, fundamental to understand the effects of thermal stress on different organisms (Chevin et al., 2010; Jørgensen et al., 2022). A major effect of severe heat stress is the denaturation of the three‐dimensional structure of macromolecules such as proteins and cell membranes, leading to function loss, metabolic failure and, ultimately, cellular and organism death (Kokura et al., 2016; Lepock, 1997). To face heat stress, the cellular machinery may activate multiple pathways increasing the production of proteins to limit or repair cell damage (Richter et al., 2010). The best‐known examples of proteins involved in this response are the heat‐shock proteins (HSP). These proteins perform several functions essential to survival; they ensure the correct refolding of denatured proteins, prevent harmful protein aggregation (due to the association of misfolded proteins), and participate in the elimination of protein aggregates (Evgen'ev et al., 2007). Other pathways triggered by high temperature include that of unsaturated longer fatty acids and sterols accumulation, whose increased production helps to keep the fluidity of the cell matrix and of the membrane (Hazel, 1995). Likewise, the production of antioxidants and detoxification enzymes, such as super oxide dismutase (SOD) or glutathione peroxidase (GPx), helps reduce the accumulation of reactive oxygen species (ROS; Birben et al., 2012). Under heat stress, these molecular pathways interact composing an intricate multilevel response that, despite its complexity, must be rapidly activated to optimize survival (Perez et al., 2021). This response is expected to be particularly important in small ectotherms—such as insects—whose body temperature is highly correlated with that of their local environment (Angilletta & Michael, 2009).

**FIGURE 1:**
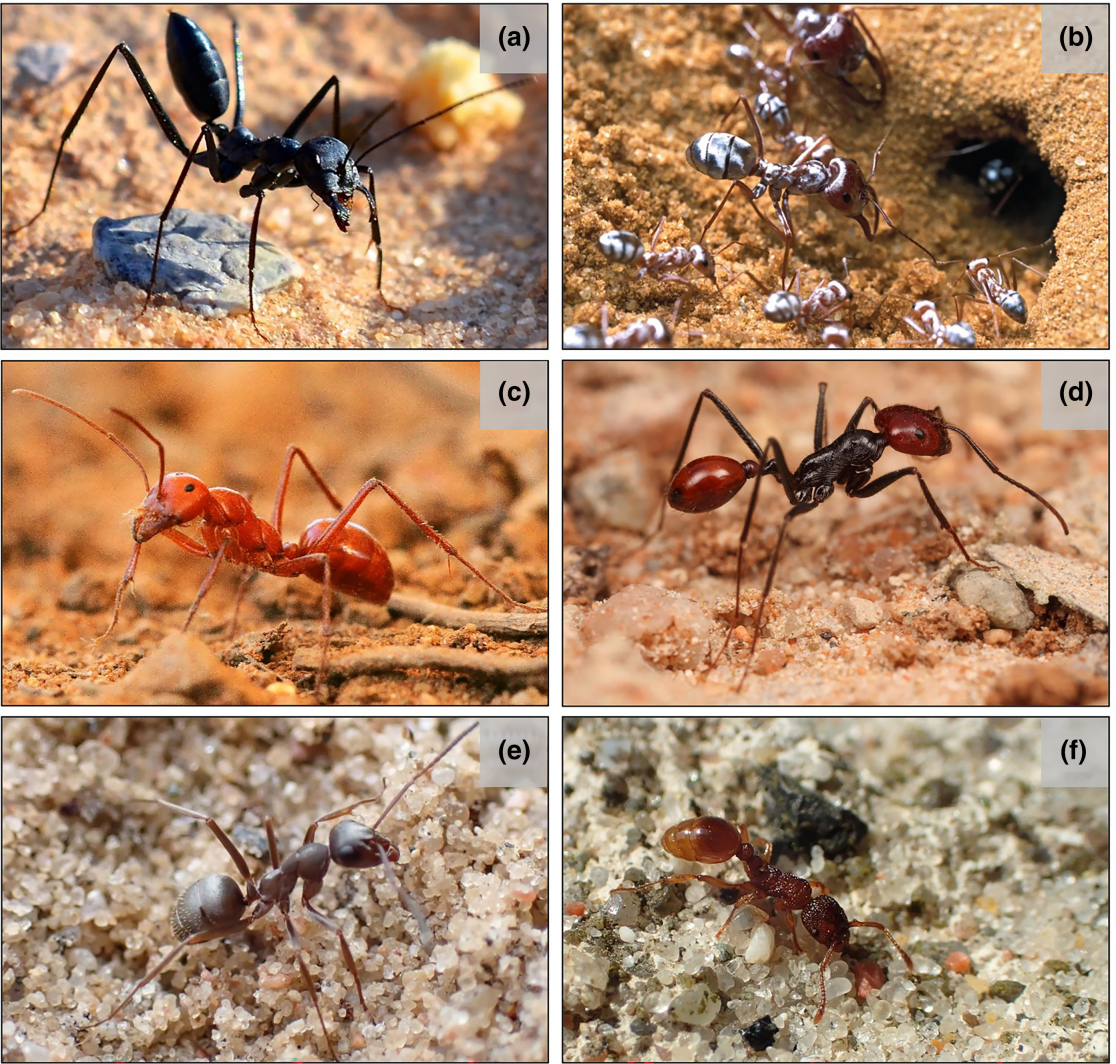
Workers of the ant species: (a) *Cataglyphis holgerseni*, (b) *C*. *bombycina*, (c) *Melophorus bagoti*, (d) *Ocymyrmex* sp., (e) *Formica fusca*, and (f) *Myrmica sabuleti*. With permission: (a) A. Kuhn, (b, e, f) Q. Willot, (c) A. Wystrach, (d) R. Duncan.

Hot deserts are among the most stressful habitats on Earth. Organisms inhabiting these regions must cope with extremely high temperatures and low humidity (Laity, 2008). These conditions have led most desert animals to adopt nocturnal or bimodal activity patterns, exploiting the cooler dawn and twilight hours to avoid the scorching heat (Costa, 1995; Marsh, 1988). Amazingly, several ant species display the exact opposite behavior; they only leave the nest during the warmest hours of the day when air and ground temperatures are close to their own lethal thermal limits (Cerdá et al., 1998; Wehner, 2020; Wehner et al., 1992). Such unusual activity pattern allows these insects to forage for heat‐struck arthropods while reducing competition and predation pressures from other desert organisms. This so‐called “thermal scavenging” behavior has appeared at least three distinct times in phylogeographically distant heat‐adapted ant genera: (A) within the *Cataglyphis* in the deserts of the northern hemisphere, (B) in some *Melophorus* species from the Australian outback, and (C) among the *Ocymyrmex* who inhabit deserts of the subtropical Africa (Figure 1; Andersen, 2007; Boulay et al., 2017; Marsh, 1985). Workers of these genera exhibit similar behavioral and morphological adaptations likely resulting from a convergent evolutionary process in response to their peculiar thermal niche (Wehner & Wehner, 2011). They can withstand body temperature exceeding 45°C during a long period of time (Christian & Morton, 1992; Turner et al., 2000; Willot et al., 2017); their legs are considerably longer relative to their body size, which maximizes the distance between their body and the burning ground and allows higher running speed reducing foraging time and enhancing forced convective cooling (Centorame et al., 2019; Sommer & Wehner, 2012); and they periodically exploit thermal refuges in shaded areas or ground elevated places to benefit from cooler spots (Marsh, 1985). Recently, the transcriptomic heat‐shock response of six *Cataglyphis* species from different microhabitats and thermal regimes has been characterized (Perez et al., 2021; Willot et al., 2018). They showed that the *Cataglyphis* heat stress response builds upon the co‐expression of gene clusters mainly involved in proteome stability, elimination of toxic residues, DNA/RNA metabolism, and the maintenance of cell integrity through membrane modification and cytoskeletal rearrangements. Whether such molecular adaptations/pathways to cope with heat stress are genera‐specific or convergently evolved in different desert ants remains however unknown.

Herein, we investigated the thermal tolerance and the transcriptomic response to heat stress of four species of desert ants belonging to the genera *Cataglyphis*, *Melophorus*, and *Ocymyrmex* (Figure 1). Additionally, to explore convergent and specific adaptations to heat stress, we compared the heat‐shock response found in these desert ants with that of related outgroup temperate species from the Formicinae (same subfamily as *Cataglyphis* and *Melophorus*) and Myrmicinae (same subfamily as *Ocymyrmex*). We first measured their heat tolerance by assessing the survival rate of workers at increasing temperatures and established the species' thermal limit. Then, we identified genes involved in the heat‐shock response by quantifying the differentially regulated transcripts between heat‐stressed and control workers. Finally, we performed cross‐species comparisons using differential gene expression and survival results to identify whether any convergence could be observed in the heat‐shock response of desert species. We show that desert ants benefit from two main molecular strategies to deal with high temperatures. The first is a constitutive high expression of heat stress genes; this response is associated with individuals' survival decreasing rapidly after reaching their thermal limit. The second was a reactive gene expression regulation in response to increasing temperatures that was associated with a wider variation in individual survival capacity above the species' thermal limit. Additionally, unlike temperate species, desert ants differentially expressed fewer transcripts even at considerably higher temperatures, indicating this is a key molecular adaptation to withstand heat stress.

## 2 MATERIALS AND METHODS

### 2.1 Ant sampling and rearing conditions

We studied six ant species from different locations. Four from arid and sandy deserts: *Cataglyphis bombycina* (Morocco), *C*. *holgerseni* (Israel), *Ocymyrmex robustior* (Namibia), and *Melophorus bagoti* (Australia); and two from a temperate region: *Formica fusca* and *Myrmica sabuleti* (Belgium). The four desert species can be considered ecologically equivalent (Perez & Aron, 2020). They inhabit sandy desert environments with mean annual temperature (AMT) above 21°C, and warmest month recorded (MaxT) ranging from 32.3 to 42.3°C (Table 1). As for the two temperate species, they inhabit temperate areas with AMT of 10.2°C and MaxT of 22.8°C. The three genera of heat‐adapted ants (*Cataglyphis*, *Ocymyrmex*, and *Melophorus*) were chosen to account for independent origins of heat stress adaptation in ants across the major deserts in the globe. Moreover, to account for intragenus variation, we included two species of desert ants from the same genus (*C*. *bombycina* and *C*. *holgerseni*). These *Cataglyphis* have shown distinct survival and expression patterns in a previous study (Perez et al., 2021). Finally, the two temperate ant species, *Formica fusca* and *Myrmica sabuleti*, were chosen as comparative nondesert outgroup genera, as they belong, respectively, to the Formicinae (same subfamily as *Cataglyphis* and *Melophorus*) and the Myrmicinae (same subfamily as *Ocymyrmex*). Colonies of desert species studied are headed by a single queen (monogyny) and queens are polyandrous (multiple mated), except for *O*. *robustior* in which queens are strictly monandrous (de Pletincx & Aron, 2020; Lecocq de Pletincx et al., 2019, 2021; Leniaud et al., 2015). The two temperate species studied are facultatively polygynous (with a single or several reproductive queens per colony); *F*. *fusca* is facultatively polyandrous, and no information about queen mating frequency is available for *Myr*. *sabuleti* (Bargum & Sundström, 2007; Elmes & Keller, 1993).

**TABLE 1:** Main thermal characteristics of species' natural habitats.

Species	Annual mean temperature	Mean diurnal range	Mean temperature of warmest quarter	Maximum temperature	Coordinates
*Cataglyphis bombycina* (Morocco)	21.0	17.2	30.8	42.3	30°33′22″ N −5°83′83″ E
*Cataglyphis holgerseni* (Israël)	22.9	13.1	29.6	37.3	30°41′22″ N 35°14′14″ E
*Melophorus bagoti* (Australia)	21	16.3	28.2	37.3	−23°50′35″ N 133°57′18″ E
*Ocymyrmex robustior* (Namibia)	21.1	16.3	23.6	32.3	−23°33′45″ N 15°2′31″ E
*Myrmica sabuleti*/*Formica fusca* (Belgium)	10.2	8.2	17.1	22.8	50°38′41″ N 4°14′22″ E

*Note*: Temperature values are in °C. The climatic data were obtained from the WorldClim database using a resolution of 30 arc‐seconds.

We excavated 4–6 colonies for each species at sampling sites and brought them back to the laboratory accompanied by one queen, brood, and workers. The colonies were kept under steady conditions: mean temperature of 25°C (± 1°C) for desert species and 18°C (± 1°C) for temperate species, a light–dark cycle of 12 h/12 h, and relative humidity between 30% and 40% for all ants. These temperatures correspond to average temperatures measured on the field at 50 cm deep in the nest, for two species *C*. *bombycina* and *F*. *fusca*. Environmental conditions during this adaptation period were established based on the species optimal parameters for laboratory brood rearing (personal observations). Ants were fed with a sugar solution provided ad libitum, and sliced mealworms that were provided three times per week. Colonies were maintained under these conditions for one month prior to the beginning of the assays to avoid short‐term gene expression alterations related to the sampling and transportation of the colonies. Only adult workers with a highly sclerotized and melanized exoskeleton (i.e., likely born in the field) were used in assays to avoid potential developmental alterations in individuals not raised in natural desert conditions.

### 2.2 Heat tolerance assays

Workers heat tolerance was determined using a heat stress assay, as detailed by Perez et al. (2021) and Willot et al. (2017). For each species, six groups (*n* = 6) of 10 randomly selected workers (from 4 to 6 colonies per species) were placed in glass tubes—totaling 60 workers—and used in the heat tolerance assays. Because body size influences heat tolerance due to differences in relative water loss (Cerdá & Retana, 2000; Clémencet et al., 2010; Hood & Tschinkel, 1990), a wet cotton ball was added in the testing tubes to reduce ant desiccation and its effect on specimens' survival during the tests. The tubes were immersed in a water bath (SW22, Julabo GmbH) kept at constant temperatures of 39, 41, 43, 45, 47, 49, or 51°C. The temperature was monitored using 0.075‐mm‐diameter thermocouples (Type K Thermocouple [Chromel/Alumel], RS Components Ltd) connected to a digital thermometer (RS Pro RS52 Digital Thermometer, RS Components Ltd) and placed within the testing tubes. Percent survival was recorded after 3 h; workers were identified as moribund once they lost their locomotor ability (i.e., muscular paralysis; Roeder et al., 2021). For each species, we measured two indicative parameters of heat tolerance: (A) the upper thermal limit (UTL_3h), the temperature below which the survival rate is significantly different from 100% (ANOVA test followed by a Tukey's test; *p* < .05), and (B) the median lethal temperature (LT50_3h), the temperature at which survival probability equals 50%. LT50_3h was estimated using a simple logistic regression of death probability as a function of the temperature. The LT50_3h, and its 95% confidence intervals, were compared between species using the ratio test (Wheeler et al., 2006), implemented in the *ratio_test* function from the *ecotox* package (Hlina et al., 2021).

### 2.3 RNA‐Seq library preparation and sequencing

For each species, we sequenced the total RNA of four replicates per treatment (*n* = 4). In the heat stress (HS) treatment, workers were exposed to their UTL_3h for 3 h; in the nonheat stress (NHS or control) treatment, workers were maintained in the glass tubes for 3 h at 25 or 18°C, for desert and temperate species, respectively. Each sample replicate contained 10 pooled workers randomly chosen among the different colonies of a species (4–6 colonies per species), totalizing 40 workers per treatment across four replicates. Ants were immediately frozen after treatments and stored at −80°C until RNA extraction. Workers were pooled to avoid individual and colony biases expression responses. Since heat stress may affect multiple body tissues, we used entire bodies for expression analyses. RNA extractions, RNA quality assessments (determined using Bioanalyzer), RNA‐seq library preparations, and sequencing were performed by BGI Tech Solutions. Total RNA was extracted using Trizol (Invitrogen) according to the manufacturer's instructions (EDTA 250 mM was added to *Formica fusca* extractions to avoid non‐enzymatic RNA degradation; Valles et al., 2012). Sequencing was performed using an Illumina HiSeq 4000 System. About 25 million single reads of 50 bp in length were generated per sample replicate. We additionally sequenced for each species a pool of workers (HS and NHS), eggs, and pupae using a HiSeq X Ten System that was used to assemble the species reference transcriptomes. About 90 million paired reads of 150 bp in length were obtained for each of these species pools.

### 2.4 Transcriptome assembly and differential expression

Transcriptome assembly and differential expression analyses were performed independently for each species. The quality of all sequenced reads was estimated using FastQC v0.11.7 (Andrews, 2010). We used the Trinity v2.8.4 pipelines (Grabherr et al., 2011) with the sequenced dataset from the pool of workers, eggs, and pupae for transcriptome assemblies. To increase assembly efficiency, reads were first digitally normalized (20× coverage) through computational coverage systematization (Brown et al., 2012). Then, two assembly strategies were used depending on the species: a full de novo and a reference‐guided assembly. For the two *Cataglyphis* species and for *Formica fusca*, we generated both, the full de novo and the reference‐guided assemblies. The reference‐guided approach for the *Cataglyphis* was based on the genome of *Cataglyphis hispanica* (assembly ASM419527v1; Darras et al., 2022) and that of *F*. *fusca* in the genome of *Formica exsecta* (assembly ASM365146v1; Dhaygude et al., 2019). These two independent assemblies were then concatenated. For *Ocymyrmex robustior*, *Melophorus bagoti*, and *Myrmica sabuleti*, only the full de novo assembly was performed due to the lack of a close reference genome. In all cases, transcripts redundancy was initially removed with CD‐Hit v4.8.1 (Huang et al., 2010) using the threshold of 95% nucleotide similarity, then Corset v1.08 (Davidson & Oshlack, 2014) and Lace v1.13 (Davidson et al., 2017) were used to cluster transcripts into superTranscripts, which further reduced transcript redundancy and improved posterior gene expression counts (Davidson et al., 2017). Assemblies were then annotated with Annocript v2.0.1 (Musacchia et al., 2015) in tandem with the UniProt Reference Clusters (UniRef90) and the UniProtKB/Swiss‐Prot (Suzek et al., 2015) databases (March 2019 versions). Only transcripts potentially encoding proteins—based on ORF estimation or blast results—were retained in the final reference transcriptome assemblies. All the parameters used in the assembly and annotation pipelines were the programs' suggested defaults unless otherwise stated. Assessments of assemblies' quality were obtained with BUSCO v5.3.2 (Simão et al., 2015), against the Hymenoptera (odb10) database, and with QUAST v5.0.2 (Gurevich et al., 2013).

For the differential gene expression analyses, reads from ants of the HS and NHS treatments were aligned to the reference transcriptome and counted using Salmon (Patro et al., 2017). The transcripts that were differentially expressed between treatments were identified using the edgeR (Robinson et al., 2009) and the DESeq2 (Love et al., 2014) pipelines. To increase stringency and reduce the rate of false positive results, the differentially expressed transcripts were selected based on the overlapping results of both analyses (i.e., overlapping results from edgeR and DESeq2) and using a cut‐off of mean absolute log_2_‐fold change ≥2 between treatments and FDR ≤ 1e‐3 (as suggested in the Trinity pipeline for de novo transcriptomes). We then computationally compared the list of differentially expressed transcripts between each species pair based on their UniRef90 gene name annotation using R (*stringr* library). Only unique, nonredundant, and meaningful gene annotations (i.e., genes whose annotation did not contain “uncharacterized protein”) were included in this comparison and in the statistical tests. After this computational comparison of gene names, the final list of overlapping genes was manually curated to avoid partial or redundant annotation matches. The significance of common differentially expressed genes between species pairs was assessed without manual curation using 10,000 random samplings comparisons based on the transcriptome datasets, that is, the same number of DET was randomly sampled from the transcriptome of each species and compared with another species randomly sampled in the same manner. These comparisons were repeated 10,000 times per species pair and a cut‐off *p*‐value < .01 of finding the same number of common DET by chance was used.

### 2.5 Functional analyses of the differentially expressed genes

To test whether gene ontology (GO) terms for biological processes (BP) were enriched among the differentially expressed transcripts, we kept only transcripts displaying nonzero expression in the treatment groups (i.e., for *C*. *bombycina*: 29,965 transcripts; for *C*. *holgerseni*: 26,769 transcripts; for *Mel*. *bagoti*: 38,726 transcripts; for *O*. *robustior*: 45,701 transcripts; for *M*. *sabuleti*: 84,784 transcripts; and for *F*. *fusca*: 62,416 transcripts). We used the “*weight01*” algorithm with the Fisher enrichment test in the R package TopGO (Alexa & Rahnenfuhrer, 2016) for the enrichment test, and significance was evaluated based on an adjusted alpha level of .01 to obtain only highly significant enriched terms. For summary visualization of enriched GO terms in desert and nondesert species, we used the Revigo server and the *Drosophila* database (Supek et al., 2011).

For each species, the function of all differentially expressed transcripts was manually checked and categorized according to their metabolic and cellular role from which we selected the nine most frequent categories: Apoptosis, Autophagy, Chaperoning, Cytoskeletal rearrangement, DNA/RNA metabolism, Lipid metabolism, Proteolysis, ROS elimination, Sugar metabolism, Other and Uncharacterized (i.e., genes of unknown function or not annotated). Finally, we assessed gene involvement in specific biochemical and metabolic pathways using the Kyoto Encyclopedia of Genes and Genomes (KEGG), via the KEGG Automatic Annotation Server (KAAS; Moriya et al., 2007).

### 2.6 Relative expression levels of heat‐shock proteins

We filtered transcripts in the reference transcriptome that were annotated to the heat‐shock protein genes HSP70 and HSP90 using a custom script (Find_Genes.py) based on representative search terms annotation (i.e., *Hsp70*, *Hsp 70*, *Heat shock protein 70*, *Hsp90*, *Hsp 90*, *Heat shock protein 90*). For each of these transcripts, we then plotted their normalized expression count in TMM from all heat‐stressed and nonheat‐stressed replicates for comparison of their relative expression levels between conditions.

### 2.7 Orthologous and new genes identification

We used TransDecoder v5.5.0 (Haas et al., 2013) to convert transcriptome ORF regions to amino acid sequences. Using OrthoFinder v2.5.4 (Emms & Kelly, 2019) with default parameters, we then estimated orthology among the transcriptomes of all species and generated the species phylogenetic tree using MAFFT for sequence alignment and FasTree for tree estimation. Orthogroups containing at least one transcript differentially expressed were considered as a differentially expressed orthogroup. We classified the orthogroups into five categories filtering the OrthoFinder resulting table: I—orthogroups containing representative transcripts from all ants, II—orthogroups containing representative transcripts only in Myrmicinae ants, III—orthogroups containing representative transcripts only in Formicinae ants, IV—orthogroups containing representative transcripts only in the two *Cataglyphis* species, accounting for intra genus similarities, and V—species‐specific orthogroups. Transcripts within orthogroups in categories IV and V were considered as taxonomically restricted genes, while categories I, II, and III were composed of conserved orthologous. For each species, only the relevant categories were considered. Fisher's exact test was used to test whether the proportion of genes in each of these categories diverged significantly between the differentially expressed transcripts and the entire species transcriptome.

## 3 RESULTS

### 3.1 Desert ants show two survival patterns aligned to two distinct gene expression regulation responses under heat stress

**FIGURE 2:**
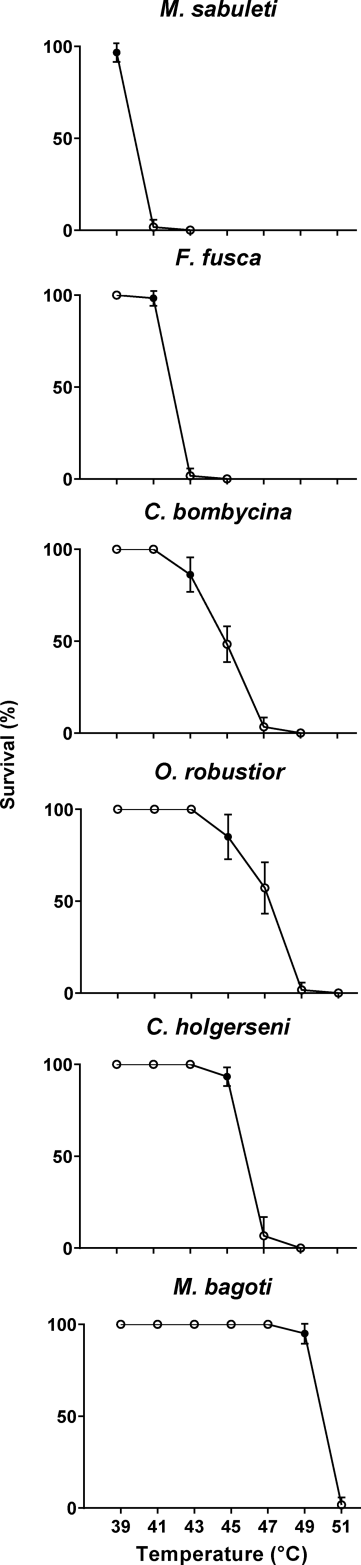
Percent survival of ant workers during heat stress assays. The ants (60 workers from 4 to 6 colonies per species) were exposed to increasing temperatures (39, 41, 43, 45, 47, 49, or 51°C) for 3 h; their percent survival was then measured. Workers were categorized as moribund when reaching muscular paralysis. For each species, UTL is indicated by a filled circle (•).

In the heat tolerance assays, the median lethal temperature after 3 h of exposure (LT50_3h) varied significantly across all species (Figure S1). It was 39.91°C (CI 95%: ± 0.16) for *Myr*. *sabuleti*; 42°C (± 0.17) for *F*. *fusca*; 44.83°C (± 0.18) for *C*. *bombycina*; 46°C (± 0.16) for *C*. *holgerseni*; 46.8°C (± 0.16) for *O*. *robustior*; and 49.83°C (± 0.15) for *Mel*. *bagoti*. Consistently, the temperate ants were also the least heat‐tolerant species according to their upper thermal limit temperatures (UTL_3h). The temperature below which the survival rate was significantly different from 100% after 3‐h exposure (upper thermal limit, UTL_3h) in *Myr*. *sabuleti* was 39°C and in *F*. *fusca* 41°C, while for the desert species, it varied from 43°C (in *C*. *bombycina* and *O*. *robustior*) to 49°C in *Mel*. *bagoti*, the most heat‐tolerant ant species in our analyses. *C*. *holgerseni* had an intermediate UTL_3h for desert species of 45°C. For all species, the survival ratio markedly dropped from around 100% to 0% within a two‐degree temperature increase interval (Figure 2), except for the Sahara ant—*C*. *bombycina*—and the Namib ant—*O*. *robustior*—whose survival gradually decreased within a wider temperature range of six degrees. The survival ratio observed for all species during assays follows in S22 at Dryad (10.5061/dryad.sn02v6x9h).

Reference transcriptome sizes ranged from 38,726 in *Mel*. *bagoti* to 84,784 in *Myr*. *sabuleti* (Figure 4b, major quality parameters are given in Table 2). The cause for such variation in transcriptome size among species is unknown; it might stem from the presence of multiple isoforms, the nature of the transcripts (CG or AT‐rich), or still the quality of the reference genome used. Assembly strategy (with or without the reference genome) did not relate directly to the total number of transcripts, as species with a reference genome did not necessarily have fewer transcripts. Instead, transcripts were likely more complete—with higher N50 values and more complete BUSCOs (Table 2)—when using the reference genome during assembly.

**TABLE 2:** Major summary quality parameters of the reference transcriptome assemblies.

	*Cataglyphis bombycina*	*Cataglyphis holgerseni*	*Melophorus bagoti*	*Ocymyrmex robustior*	*Formica fusca*	*Myrmica sabuleti*
Number of transcripts	41,912	44,525	38,726	45,701	62,416	84,784
Total length (pb)	115,117,532	112,126,054	85,836,189	91,769,795	146,813,706	139,366,828
N50	4446	4153	3929	3738	4507	3123
BUSCOs	C:93.5% [S:26.9%, D:66.6%], F:2.7%, M:3.8%	C:92.9% [S:28.2%, D:64.7%], F:2.8%, M:4.3%	C:85.9% [S:34.2%, D:51.7%], F:5.0%, M:9.1%	C:85.3% [S:25.7%, D:59.6%], F:5.6%, M:9.1%	C:95.9% [S:29.4%, D:66.5%], F:1.2%, M:2.9%	C:86.7% [S:23.6%, D:63.1%], F:5.4%, M:7.9%
Blast hits to Swiss‐Prot	24,805	25,225	21,369	27,151	30,795	30,858
Blast hits to UniRef90	35,155	36,537	33,296	40,188	48,761	61,977
Transcripts with at least one blast hit	35,416	36,951	33,329	40,229	48,804	62,037

*Note*: BUSCOs parameters are based on an N of 5991 orthologs: C—complete; S—single copy; D—duplicated; F—fragmented; M—missing.

**FIGURE 3:**
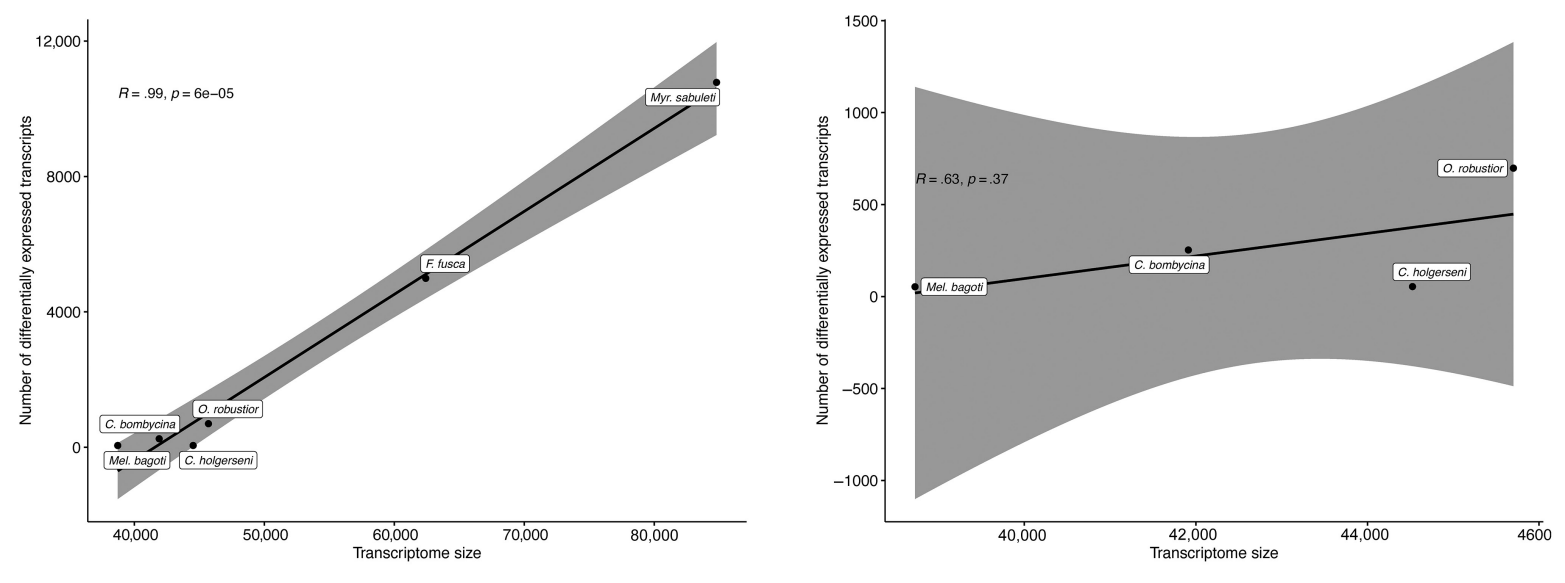
Correlation between total transcriptome size and the number of differentially expressed transcripts under heat stress. Left: all species included. Right: only desert ant species included. Correlation method Pearson, significance at *p* < .05.

**FIGURE 4:**
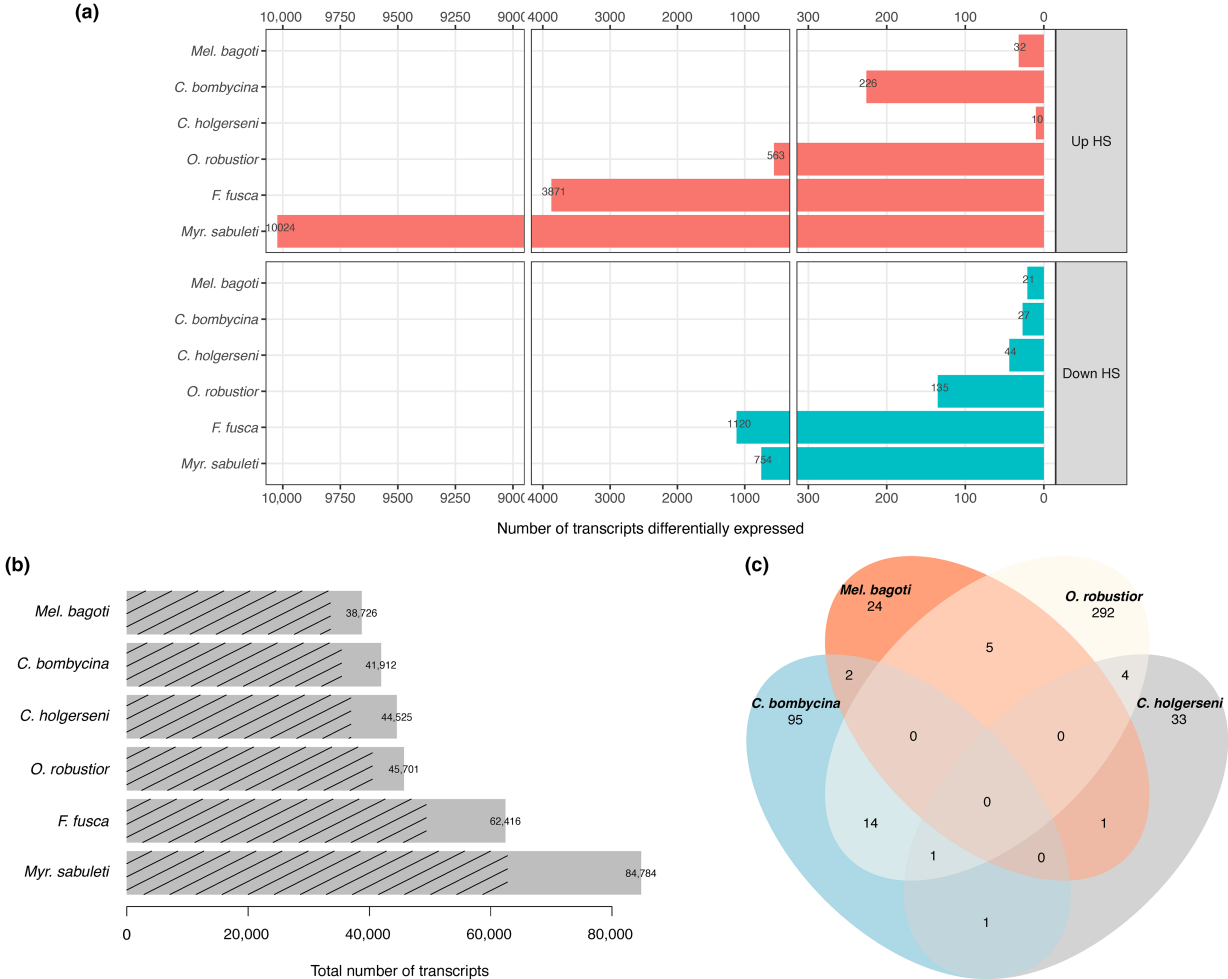
Transcriptomic analyses summary. (a)—Number of differentially expressed transcripts between heat‐stressed and nonheat‐stressed conditions for each species tested. Up HS transcripts refers to transcripts upregulated in the heat‐stressed samples when compared to the nonheat‐stressed samples, while Down HS refers to transcripts downregulated in heat‐stressed samples (i.e., upregulated in nonheat‐stressed samples in comparison with heat‐stressed ones). (b)—Reference transcriptome sizes. Dashed lines show the proportion of transcripts with at least one significant blast hit. (c)—Number of transcripts differentially expressed and annotated in common among desert species. Only the overlap between *Cataglyphis bombycina* and *Ocymyrmex robustior* was significant at *p* < .01 random sampling test. Overlap considered only unique, nonredundant, and meaningful gene annotation names, explaining the small overlapping values reported in the figure. For the sake of clarity, overlapping results are shown only for desert species. All species comparisons are given in Table 3.

**FIGURE 5:**
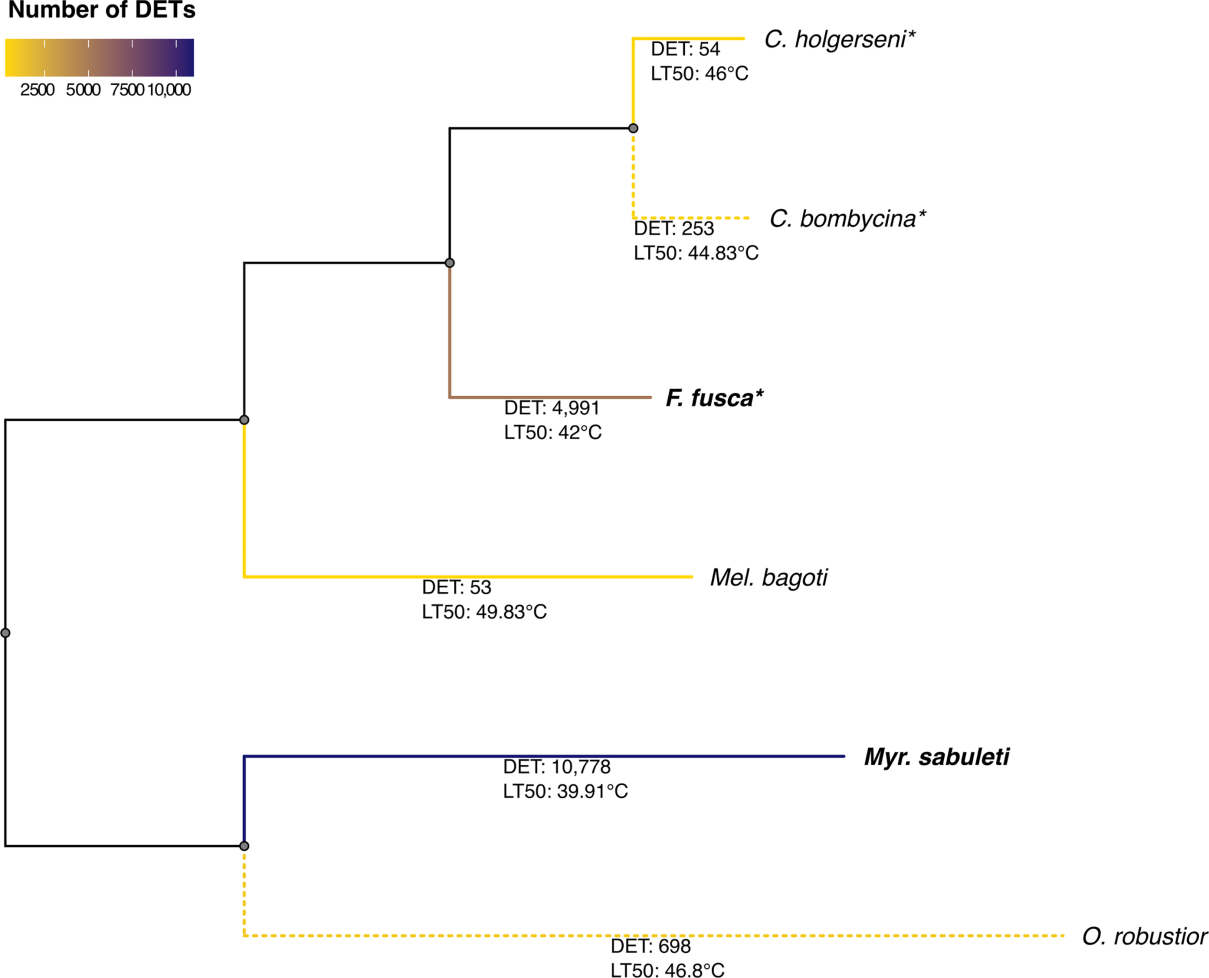
Phylogenetic tree of the studied ant species was estimated using orthologous proteins. Branch colors correspond to the number of differentially expressed transcripts (DET) identified for that species between heat‐stressed and nonheat‐stressed samples. The exact number of DET is reported below each species branch along with the LT50. Gray lines represent internal nodes with no values. Dotted lines indicate species showing a gradual decrease in survival ratio within a six‐degree temperature interval while straight lines refer to species whose survival decline from 100% to 0% within a smaller temperature range of two degrees. Ant species from temperate areas are shown in bold. Species with a reference genome used in the transcriptome assembly are indicated with an *.

The number of transcripts differentially expressed between the HS and NHS conditions ranged from 53 in *Mel*. *bagoti* to 10,778 in *Myr*. *sabuleti* (Figure 4a; complete lists of DET in each species and their annotations are provided in Files S2–S13). Based on the range of differential gene expression observed across the species, we grouped their responses into three responsive profiles that were not phylogenetically conserved (Figure 5): the first comprised *Mel*. *bagoti* and *C*. *holgerseni*, both species showing weak heat stress expression responses with only 53 DET in 38,726 transcripts (0.14%) and 54 DET in 44,525 transcripts (0.12%), respectively; the second included *C*. *bombycina* and *O*. *robustior*, which had a medium heat stress expression response of 253 DET in 41,912 transcripts (0.60%) and 698 DET in 45,701 transcripts (1.53%), respectively; the third group contained *F*. *fusca* and *Myr*. *sabuleti* which had stronger heat stress responses and differentially expressed 4991 in 62,416 transcripts (8%) and 10,778 in 84,784 transcripts (12.71%), respectively. We found a highly significant correlation between transcriptome size and the number of transcripts differentially expressed in response to heat stress across all species (*Pearson R* = .99, *p* = 6e−05, Figure 3). However, when only desert species were considered, this correlation was lost (*Pearson R* = .63, *p* = .37, Figure 3). This suggests that the difference in the number of DET between desert and temperate species is at least partly explained by differences in their transcriptome sizes. However, the difference between transcriptome sizes and the number of DET were of distinct magnitudes; temperate species showed a minimum sevenfold increase in gene expression activity during assays in comparison with desert ants (Figure 4a), while the maximum transcriptome size difference was only onefold greater (Figure 4b). Thus, we argue changes in transcriptome assembly alone could not account for all the differences in DET observed.

In conclusion, desert ants displayed fewer gene expression changes in response to thermal stress than temperate species and transcriptome size variation alone cannot account for all the observed differences. Furthermore, among desert ants, wider gene expression alterations in response to heat stress were linked to larger variation in workers' survival: species with a more intense expression response (i.e., *C*. *bombycina* and *O*. *robustior*; Figure 4) displayed a more gradual decrease in survival ratio (Figure 2)—dropping from 100% to 0% survival within a six‐degree temperature interval—while species with weak heat stress expression response (i.e., *C*. *holgerseni* and *Mel*. *bagoti*) showed a marked reduction in survival within a smaller temperature range of two degrees. In temperate species, survival also decreased rapidly within a two‐degree temperature increase; however, in contrast to desert ants, many genes were differentially expressed (Figure 5).

### 3.2 Fewer gene expression alterations in response to heat stem from high constitutive levels of heat stress‐related genes in desert ants

**FIGURE 6:**
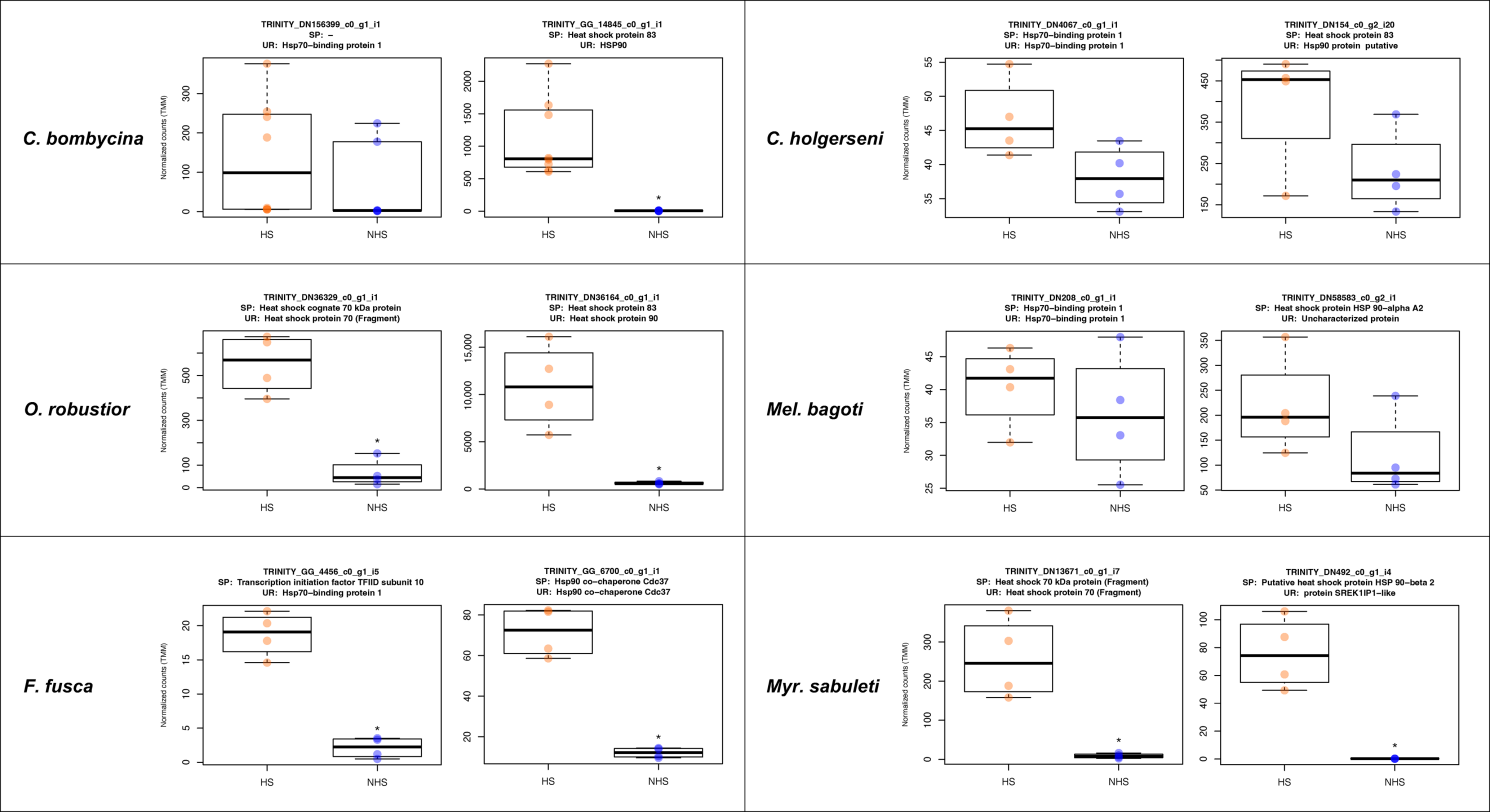
Expression of heat stress proteins 70 and 90 related transcripts in heat‐stressed and nonheat‐stressed treatments. Expression levels are shown as TMM normalized read counts. The transcripts shown represent one related transcript from each HSP (70 or 90) that was either differentially expressed or had the highest expression in the referred species, that is, the most expressed isoform. HS—samples heat stressed; NHS—nonheat‐stressed control samples; SP—UniProtKB/Swiss‐Prot annotation; UR—UniRef90 database annotation. * indicates the transcript is significantly differentially regulated between treatment conditions (log_2_‐fold change ≥ 2 between treatments and FDR ≤ 1e‐3). Nondesert adapted species are shown in the bottom.

To further investigate whether the different expression patterns found among desert ants were due to a lack of expression of heat stress related genes, or instead, to a distinct timing regulation of these genes, we focused on the expression pattern of admittedly known heat stress‐related genes—the heat‐shock proteins 70 and 90 (HSP70 and HSP90)—and inspected the normalized counts of their related transcripts (in TMM) in HS and NHS replicates. The expression plots of these transcripts in all species are reported in Figures [Link], [Link], [Link], [Link], [Link], [Link]. Although expression counts of desert ants HSP70 and HSP90 transcripts under heat stress and nonheat stress conditions were only significantly differentially regulated in *C*. *bombycina* and *O*. *robustior*, these genes were still expressed in *C*. *holgerseni* and *Mel*. *bagoti* (Figure 6). In the two latter species, however, the expression of HSP70 and HSP90 in the control group (NHS ants) was already elevated, which explains the lack of significant differences between the two groups. This indicates that desert species with fewer gene expression alterations (i.e., *C*. *holgerseni* and *Mel*. *bagoti*) have a constitutive upregulation of HSP70 and HSP90 and do not significantly change their expression levels when exposed to increasing temperatures, unlike *C*. *bombycina* and *O*. *robustior*.

These results support the hypothesis that the reduced number of differentially expressed genes under heat stress in *C*. *holgerseni* and *Mel*. *bagoti* stems from a constitutive upregulation of heat stress‐related genes. Therefore, the distinct expression patterns observed in desert ants would reflect a difference in timing regulation of heat stress‐related genes with *C*. *holgerseni* and *Mel*. *bagoti* constantly keeping a regular expression of these genes, while *C*. *bombycina* and *O*. *robustior* alter their expression in response to heat stress.

### 3.3 Molecular pathways responsive to heat stress in desert ants with greater gene expression changes are also activated in temperate species

**FIGURE 7:**
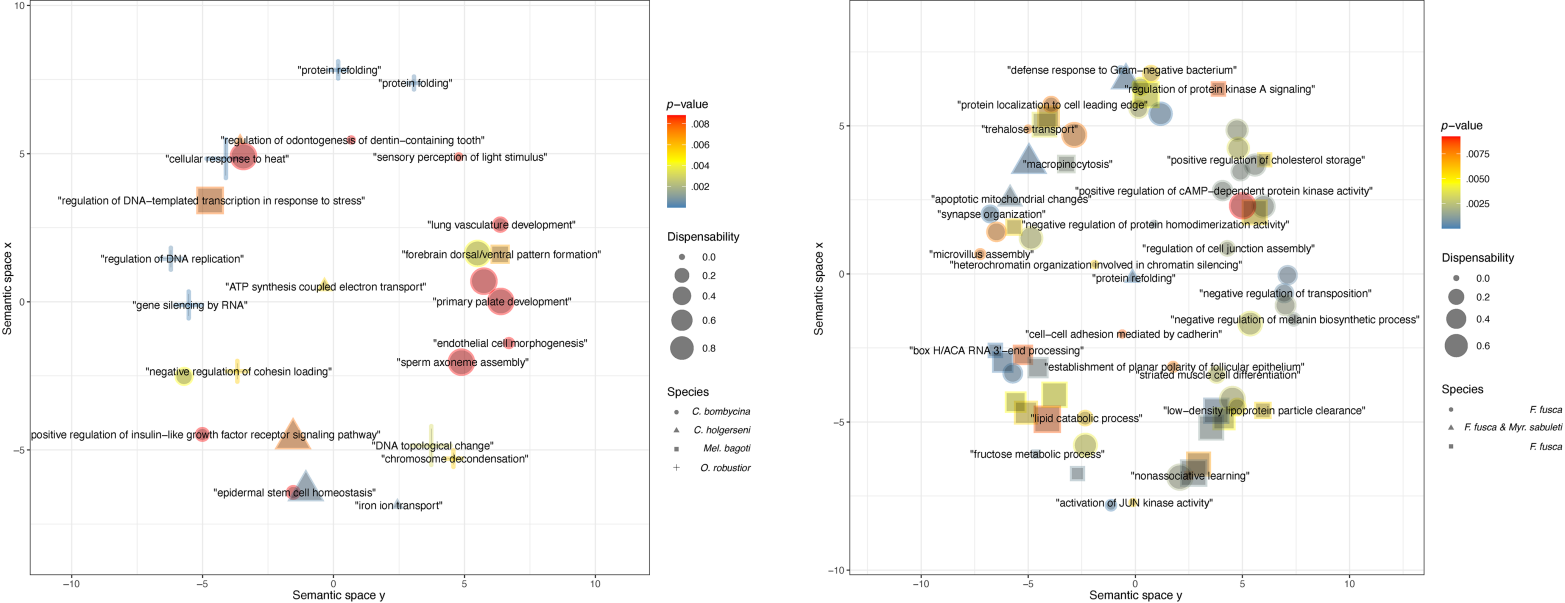
Summary of the enriched GO terms based on terms similarity as indicated by REVIGO. On the left is represented all GO terms enriched in desert ants, on the right enriched terms in temperate species. Colors indicate enrichment *p*‐values, form size the dispensability of the term within the considered semantic space, and form shape shows in which species the term was significantly enriched.

**FIGURE 8:**
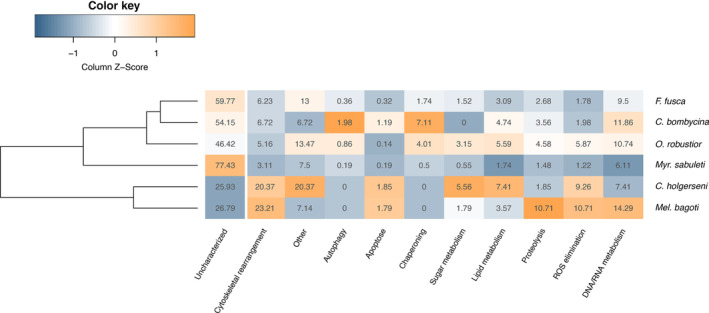
Relative proportion of differentially expressed transcripts involved in heat stress related functional categories. “Other” represents genes involved in any other biological function different from the ones detailed in the figure. “Uncharacterized” genes are shown for reference in the first column. Data were clustered according to their Euclidean distances and not according to species phylogenetic relationship. Color is scaled by columns, that is, for each column largest values are indicated in dark orange and the smallest values in dark blue.

Among desert ants, only *C*. *bombycina* and *O*. *robustior* had a significant number of DET in common (Figure 4c). When including the ant species from temperate regions in the comparisons, however, there was a significant number of DET in common among all species except *C*. *holgerseni* and *Mel*. *bagoti* (Table 3). The list of genes found to be commonly differentially expressed across species follows in File S20. Similarly, from the GO analyses (Table S1, Figure 7), we found that terms associated with protein folding (“Protein folding,” “Protein refolding,” and “*de novo* protein folding,” respectively GO:0006457, GO:0042026, and GO:0006458) and cellular response to heat (GO:0034605) were commonly enriched in *O*. *robustior*, *Myr*. *sabuleti*, and *F*. *fusca*. In *C*. *bombycina*, although several *hsp* genes were upregulated (File S2), protein folding GO terms were not significantly enriched using our adjusted *p*‐value cut‐off at <.01 (“Protein refolding,” *p*‐value = .025). Between *Myr*. *sabuleti* and *F*. *fusca*, two species with a larger number of enriched GO terms (Table S1, Figure 7), biological processes involved in mitochondria homeostasis, actin or microtubules organization, lipid metabolism, cellular signaling and transduction, and DNA/RNA metabolism also overlapped. When we categorized the differentially expressed genes into the most frequent functional categories (Figure 8), instead of only focusing on significantly enriched GO terms, we see that for all species most of the characterized differentially expressed genes are commonly comprised within the same categories being the larger proportion of them involved with cytoskeletal rearrangement and DNA/RNA metabolism. Notably, *C*. *holgerseni* and *Mel*. *bagoti* did not differentially regulate any gene involved in chaperoning and autophagy. Thus, for the four species with a wider gene regulation response (i.e., the desert species *C*. *bombycina* and *O*. *robustior*, as well as the temperate species *Myr*. *sabuleti* and *F*. *fusca*), there was a conserved differential regulation involving the same genes and pathways in response to heat stress.

**TABLE 3:** Number of differentially expressed genes under heat stress common among all ant species.

	*Cataglyphis bombycina*	*Cataglyphis holgerseni*	*Formica fusca*	*Ocymyrmex robustior*	*Melophorus bagoti*	*Myrmica sabuleti*
*C*. *bombycina*	113	2	42*	15*	2	40*
*C*. *holgerseni*	0.83 (0.90)	40	13	5	1	12
*F*. *fusca*	23.5 (4.52)	8.7 (2.78)	1394	95*	12	419*
*O*. *robustior*	5.1 (2.20)	1.88 (1.35)	53.42 (6.85)	315	5	116*
*Mel*. *bagoti*	0.62 (0.79)	0.23 (0.47)	6.64 (2.42)	1.44 (1.18)	31	11
*Myr*. *sabuleti*	27.36 (4.76)	10.13 (3.01)	289.72 (14.81)	76.65 (7.96)	7.91 (2.63)	2048

*Note*: The number of differentially expressed genes shared by each species pair is shown in the upper right‐hand part of the table. Shown in the lower left‐hand part of the table are the mean numbers and the standard deviation (in parentheses) of the differentially expressed genes expected to be shared by each species pair, based on the distributions of 10,000 random iterations. The cells in gray show the numbers of unique non‐redundant genes that were differentially expressed in each species. *Significant number of genes in common (*p* < .01).

To better visualize the metabolic and biochemical pathways of heat‐shock response, we analyzed the KEGG pathways of DET (FCx2) for each species and checked the biological function of each KEGG ortholog (KO) assigned. Overall, *C*. *bombycina* (7 KO), *C*. *holgerseni* (4 KO) and *Mel*. *bagoti* (5 KO) displayed few KEGG pathway matches. *C*. *bombycina* and *C*. *holgerseni* exhibited an upregulation of glutathione synthesis through its precursor (for *C*. *bombycina*: glycine and cysteine/methionine metabolism; for *C*. *holgerseni*: cysteine‐glycine synthesis). In addition, *C*. *holgerseni* showed a match for C10–20 isoprenoids biosynthesis (M00367). In contrast, 51 KO were assigned for *O*. *robustior* including genes involved in glutathione synthesis or its precursors (K00799, K00830, or K00273), lipid metabolism [Inositol phosphate metabolism (M00130, M00131, M00132), fatty acid synthesis and elongation (M00082 and M00083), and triacylglycerol metabolism (M00089)], sugar metabolism [glucose metabolism (K01189 and K00008), trehalose synthesis (K16055 and K00963), pentose phosphate pathway (K00115 and K01623)], proline synthesis (K0086) and lysine degradation (K01435). For *F*. *fusca* and *M*. *sabuleti*, we found 88 KO and 148 KO, respectively. In both species, KO included genes involved in sugar metabolism [glycolysis (M00001) and citrate cycle (M00010)], pentose phosphate pathway (M00165 and M00004), NADP/NADPH recycling (M00172), fatty acid elongation (M00083 and M00085), tryacyl glycerol and ceramide synthesis (M00089 and M00094) respectively and methionine salvage pathway (M00034). In addition, *F*. *fusca* showed enrichment in acyl glycerol and leucine degradation (M00098 and M00036) and *Myr*. *sabuleti* in inositol phosphate metabolism and ketone synthesis (M00130 and M00088). Overall, these results indicate the potential relevance of energetic pathways in the ants heat stress response.

### 3.4 Greater gene expression changes are associated with increased participation of taxonomically restricted genes in the heat stress response

As documented above, cross‐species comparisons of gene annotation and functional categories showed that conserved genes and pathways are largely involved in the heat stress response. Nevertheless, our results also revealed several uncharacterized genes that were differentially regulated suggesting that new genes may also play a role in heat adaptation (Figure 8). To further investigate this issue, we accessed the transcripts orthology based on their protein sequence (using OrthoFinder) and considered lineage‐specific orthogroups as taxonomically restricted, hence, new genes. From the 292,232 protein‐coding genes analyzed across all 6 species studied, 266,366 (91.1%) were assigned to at least one of the 34,135 orthogroups identified. Species‐specific orthogroups accounted for 9178 (26.9%) of all orthogroups and contained 29,645 genes (10.1% of the total), while 8753 orthogroups contained all species and only 321 were single copies (Table 4). Compared with genomic orthology estimations (Darras et al., 2022), the transcriptomic‐based orthology analyses resulted in more species‐specific and fewer single‐copy orthogroups, which can be expected since transcriptomic data might include multiple isoforms per gene (see BUSCO duplication ratio in Table 2). For this reason, we estimated the relevance of new genes not only based on their frequency but comparatively, by contrasting the relative proportion of transcripts in each orthogroup category between the transcriptome and among the DET. We expected that any bias in orthology estimation due to the transcriptomic nature of the data affects the transcriptome and its subsets in a similar manner. Thus, our null hypothesis was that the proportion of transcripts in each orthology category would be equivalent in the entire transcriptome and among the differentially expressed transcripts.

**TABLE 4:** OrthoFinder summary results.

	*Cataglyphis bombycina*	*Cataglyphis holgerseni*	*Melophorus bagoti*	*Ocymyrmex robustior*	*Formica fusca*	*Myrmica sabuleti*
Number of genes	46,104	46,417	37,949	44,826	58,025	58,911
Number of genes in orthogroups	43,429	43,520	34,985	41,217	53,023	50,192
Number of unassigned genes	2675	2897	2964	3609	5002	8719
Percentage of genes in orthogroups	94.2	93.8	92.2	91.9	91.4	85.2
Percentage of unassigned genes	5.8	6.2	7.8	8.1	8.6	14.8
Number of orthogroups containing species	17,645	17,924	16,689	17,837	19,473	20,662
Percentage of orthogroups containing species	51.7	52.5	48.9	52.3	57.0	60.5
Number of species‐specific orthogroups	712	859	960	1637	1748	3053
Number of genes in species‐specific orthogroups	2315	2594	2904	5099	6400	10,333
Percentage of genes in species‐specific orthogroups	5.0	5.6	7.7	11.4	11.0	17.5

**FIGURE 9:**
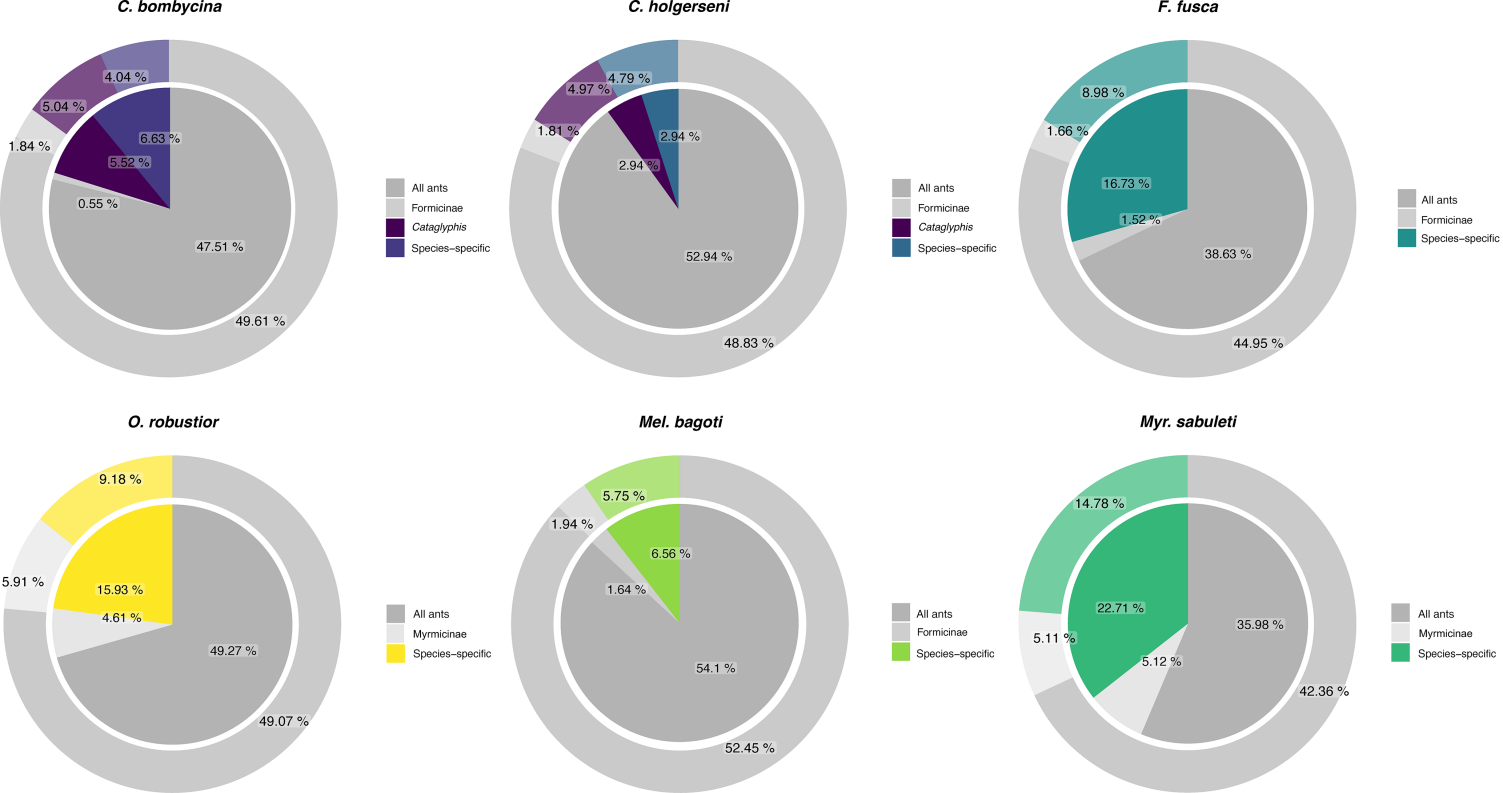
Relative proportion of transcripts in different orthologous categories. Colors highlight lineage‐specific categories while gray shades illustrate taxonomically conserved orthologous categories. Inner circles represent the proportion of transcripts in each category among the set of differentially expressed transcripts while outer circles show these proportions in the full transcriptome for comparison. Differences between the proportions observed in *Formica fusca*, *Myrmica sabuleti* and *Ocymyrmex robustior* are significant based on an alpha level of .01.

According to the OrthoFinder resulting table (File S21), we filtered orthogroups from five categories: I—orthogroups shared by all ants, II—orthogroups shared only by Myrmicinae ants, III—orthogroups shared only by Formicinae ants, IV—orthogroups shared only by the two *Cataglyphis* species, and V—species‐specific orthogroups. Categories I to III included 8753, 1055, and 324 orthogroups, respectively, and were considered as representative of taxonomically conserved genes. While categories IV and V represented taxonomically restricted genes. Category IV included 890 orthogroups. In Category V, the number of species‐specific orthogroups found for each species varied from 712 in *C*. *bombycina* to 3053 in *Myr*. *sabuleti* (all species values are given in Table 4). In species with medium and high gene regulation responses, namely *C*. *bombycina*, *O*. *robustior*, *Myr*. *sabuleti*, and *F*. *fusca*, we found an overall increase in the proportion of taxonomically restricted genes among the DET (Figure 9). This increase was significant in *F*. *fusca* (Fisher's Exact Test *p*‐value < 2.2e‐16), *Myr*. *sabuleti* (*p*‐value < 2.2e‐16) and *O*. *robustior* (*p*‐value < 4.5e‐06), but not in *C*. *bombycina*, neither in *Cataglyphis* specific nor species‐specific orthologs (*p*‐values = .732 and .086, respectively). Contrastingly, a reduction of taxonomically restricted genes among the differentially expressed transcripts was observed in *C*. *holgerseni*, and in *Mel*. *Bagoti* where only a slight increase of less than 1% in the ratio of new genes among the differentially regulated genes was found (Figure 9).

These findings show that the involvement of taxonomically restricted genes in ants' heat stress response is directly associated with the number of genes under differential expression, with species that differentially regulate more genes as temperature increases also altering the expression of a higher proportion of taxonomically restricted genes.

## 4 DISCUSSION

We characterized and compared the heat tolerance and the heat stress transcriptomic response of four ecological equivalent desert ant species from three distinct genera, and of two outgroup species living in a temperate region. From all six studied species, the red honey ant *M. bagoti* was the most heat‐tolerant, withstanding the highest temperatures ever reported in ants (Andrew et al., 2013; Johnson & Stahlschmidt, 2020; Perez et al., 2021; Solis & Bueno, 2012). However, in natural habitats, the Sahara silver ant *C. bombycina* is the species known to endure the most environmental heat, with a mean temperature in the warmest quarter of 30.8°C and a maximum temperature of 42.3°C (Table 1). An important part of *C*. *bombycina* heat resistance mechanism in their natural habitats relies on prism‐shaped hairs covering the body of workers. These hairs reflect solar radiation through total internal reflection, which limits heat absorption when the workers are exposed to sunlight (Shi et al., 2015; Willot et al., 2016). This adaptive mechanism deflects the energy associated with visible wavelengths but played no role in laboratory conditions when we measured heat tolerance through infrared warming, which could explain *C*. *bombycina* reduced thermal tolerance in our tests (see also Perez et al., 2021). Not surprisingly, the least heat‐tolerant species studied were those from temperate areas, namely *Myrmica sabuleti* and the dusky ant *Formica fusca*. Both species displayed the lowest upper thermal limit and LT50_3h values (respectively, UTL_3h: 39 and 41°C, LT50_3h: 39.9 and 42°C). In ants and other insects, there is a clear association among conditions at their microclimatic level, operative temperatures experienced by individuals, and upper‐thermal limits (Clusella‐Trullas et al., 2021; Sunday et al., 2014; Woods et al., 2015). For example, species occupying niches exposed to greater variability in environmental temperatures, such as open areas and canopies, often show increased heat tolerance (Baudier et al., 2018; Bujan et al., 2020; Kaspari et al., 2015). Our results show this association seems to also occur in strict desert thermal scavenging ants, species whose peculiar thermal niche has likely driven the evolution of higher upper thermal limits. These observations highlight the vulnerability of ectotherms, including social insects, to temperature changes (reviewed in Jørgensen et al., 2022, Martinet et al., 2021, Perez & Aron, 2020).

Temperate species differentially regulated thousands of transcripts more than thermophilic ones, showing that fewer gene expression changes under heat stress is an important adaptation of desert ants. A similar adaptative response has also been documented at the populational level; studies comparing same species populations from colder and hotter environments found that heat‐adapted populations show a reduced gene expression response under heat stress. Japanese mantis shrimp populations from temperate regions differentially regulate almost one thousand more transcripts in response to heat stress than shrimp populations inhabiting a warmer environment (Lou et al., 2019). Similarly, populations of the Chinese minnow fish from cooler regions in the north show more temperature responding genes under heat stress than southern populations (Yu et al., 2018). In the oilseed rape (*Brassica napus*), the heat‐sensitive genotype showed a larger variation in the methylation pattern of multiple genes under heat stress in comparison with the heat‐tolerant genotype (Gao et al., 2014). Since molecular damage induce the heat‐shock response (Evgen'ev et al., 2007), reduced gene expression in heat‐adapted organisms might simply reflect reduced molecular damage at higher temperatures. Selection is expected to favor proteins optimized to certain thermal environments (Fields, 2001; Fields et al., 2015; Sælensminde et al., 2009); thus, the proteome of thermophile desert species might keep protein function even at elevated temperatures delaying the molecular heat‐shock response. A recent study on *Mytilus* mussels indeed showed that small differences in habitats were sufficient to cause such protein structural alterations (Chao et al., 2020). Additionally, we found in desert ants many differentially expressed genes in response to heat stress that were related to DNA/RNA metabolism and regulation (Figures 7 and 8). This is an indication that protective mechanisms against uncontrolled gene expression, such as DNA heterochromatinization and histone aggregation, are also in place. Altogether, these results suggest that the reduced gene expression alterations observed in desert ants in comparison with temperate species likely stem from a combination of diminished molecular damage and increased gene expression control mechanisms at higher temperatures, rather than from the activation of a single gene or pathway that evolved exclusively in desert ants. In line with this, the transcriptomic response of *F*. *fusca* and *Myr*. *sabuleti* involved many of the same cellular pathways and protective mechanisms found differentially regulated in desert species. Gehring and Wehner (1995) have also observed that *Cataglyphis* and *Formica* species differed only slightly in HSP induction patterns and suggested that pre‐adaptations to heat were already present in the ancestral lineage. Our findings corroborate this observation, suggesting that temperate species have most of the molecular toolkit necessary for the heat stress response of desert ants. However, unlike the latter, temperate species co‐activate many other genes and pathways upon heat stress likely leading to an unspecific and energetically costly response.

Our results also show that desert ants evolved two adaptive strategies at the transcriptomic level to endure heat stress: (A) differentially regulating very few genes in response to heat stress, as shown by *C*. *holgerseni* and *Mel*. *bagoti*, or (B) differentially regulating several genes upon increased temperature, as observed in *C*. *bombycina* and *O*. *robustior*. In our analyses, desert species that showed stronger expression response under heat stress also displayed larger variation in temperature survival, with their survival curve gradually decreasing from 100% to 0% within a six‐degree interval (Figure 2). Contrastingly, the survival of species with fewer gene expression differences between heat‐stressed and nonheat‐stressed conditions decreased more abruptly reaching 0% within a two‐degree variation interval. In the face of these results, our hypothesis is that desert species with larger expression differences have a reactive response to heat, while desert species with fewer changes in gene expression keep a constitutive response to heat stress. Agreeingly, HSP70 and HSP90 transcripts have constant expression levels in nonheat‐stressed as well as in heat‐stressed workers of *C*. *holgerseni* and *Mel*. *bagoti* (Figure 6). This constitutive synthesis of thermoprotective molecules would provide high heat resistance, but low flexibility at their thermal limit capacity since gene expression would not be as responsive to environmental temperature changes. Interestingly, the constitutive and reactive responses were not phylogenetically conserved (Figure 6), instead they evolved convergently in different desert species genera. Similarly, in some yeast species, the expression of stress‐related genes is induced upon stress while in other species these genes evolved to be constitutively activated in spite of the stressor exposure (Tirosh et al., 2011). Other *Cataglyphis* species previously studied also showed a reactive response, like *C*. *bombycina* and *O*. *robustior*, with a maximum of 1118 DET (mean: 561 DET) accompanied by a gradual decrease in survival upon a larger temperature range (Perez et al., 2021).

Both strategies—the reactive response and the constitutive response—result in an incredible heat resistance in desert ants and could have evolved from distinct evolutionary constraints. Examples of evolutionary constraints that could lead to such differences include microhabitat differences, body size, and metabolic trade‐offs. Most *C*. *holgerseni* and *Mel*. *bagoti* foragers are larger, while *C*. *bombycina* and *O*. *robustior* workers are comparatively smaller (Figure S5). Large body sizes in desert ants could be advantageous to maintain high thermal inertia, storing more water preventing desiccation, and storing energetic resources to face cellular stress (Bouchebti et al., 2015; Johnson & Stahlschmidt, 2020; Wendt & Verble‐Pearson, 2016). It is, therefore, possible that for ant species with smaller body sizes, a reactive gene expression response would be necessary due to reduced internal storage for keeping thermoprotective molecules. It is also important to consider that these two strategies likely involve different energetic demands. The maintenance of constitutive levels of thermoprotective genes is energetically costly, however, a reactive gene expression response as temperature increases would require high amounts of energy available to be used at once to rapidly alter gene expression and to recover from the possible loss of biochemical activity from protein denaturation (Nguyen et al., 2016). Therefore, energetic trade‐offs and alterations in energetic pathways could represent important constraints to the evolution of these two strategies, as is suggested by the activation of energetic pathways in the heat stress response.

Among desert ants, in *C*. *holgerseni* and *M*. *bagoti*, we found respectively three and four differentially expressed transcripts involved in sugar energetic pathways. Interestingly, in *O*. *robustior* more than 20 transcripts were upregulated in this functional category. For example, we found genes involved in glucose synthesis (*maltase 1‐like*, *alpha‐glucosidase*, or *alpha‐amylase a‐like*) and transformation (*l glucose dehydrogenase fad quinone‐like* or *sorbitol dehydrogenase*). In addition, KEGG pathway analyses revealed that among the DET of *O*. *robustior*, there was a positive regulation of trehalose synthesis and of the pentose phosphate pathway (PPP). The former is known to enhance heat and desiccation tolerance in various organisms (Jain & Roy, 2009), while the oxidative phase of PPP is used to regenerate NADPH which is required by antioxidant enzymes such as the *glutathion S‐transferase* and the *cytochrome P450*, both also upregulated in *O*. *robustior* (Hayes et al., 2005; Pandian et al., 2020). A positive co‐regulation of sugar metabolism in response to heat stress was previously documented in *Cataglyphis* (Perez et al., 2021). Similarly, among the temperate species, many transcripts differentially expressed are involved in sugar metabolism as well as the upregulation of the PPP pathway. These results suggest that the heat‐shock response in ants requires energy consumption from the sugar metabolism. Nevertheless, in *O*. *robustior* this response was somewhat accentuated, with many of the genes and regulatory pathways involved in the antioxidative machinery along with genes involved in the trehalose synthesis being differentially regulated under heat stress.

The overlap of genes and pathways involved in the transcriptomic response to heat stress of desert and temperate ants is strong evidence of taxonomically conserved genes and pathways regulating this trait. Nevertheless, our analysis also shows an increased proportion of new genes among the DET of desert and non‐desert species when compared to the entire transcriptome (Figure 8). Only desert ants with fewer gene expression alterations were an exception to this pattern. These observations indicate that new genes are likely to be co‐expressed along with taxonomically conserved genes under heat stress, possibly because they share the same promoter regions (Kaessmann, 2010). Agreeingly, in our data, the larger the number of genes under differential expression, the larger the proportion of new genes among them. This co‐expression could offer an opportunity for gene neofunctionalization during adaptation, which is one of the most important origins of new gene fixation (Kaessmann, 2010; Werner et al., 2018). New genes are known to be particularly relevant to species' adaptative response to stress (Chen et al., 2013; Johnson, 2018; Kaessmann, 2010; Taylor & Raes, 2004). For example, in Antarctic notothenioid fishes, the ice‐biding antifreeze glycoprotein (AFGP), which originated from a functionally unrelated gene (the *pancreatic trypsinogen‐like protease2*), has been co‐opted to play a crucial adaptive role in preventing blood from freezing (Cheng & Chen, 1999). In ants, 10% to 30% of all genes in the genome are estimated to be taxonomically restricted (Wissler et al., 2013), and evidence suggests that high ratios of de novo gene formation exist within the group (Simola et al., 2013). It is therefore possible that taxonomically restricted genes are also involved in species‐specific adaptations to heat stress in desert ants. Comparative genomic studies and functional analyses should further explore these genes and their putative roles in heat stress adaptation.

Interspecies comparative transcriptomic analyses are challenging because normalization methods cannot account properly for species‐specific batch effects and the use of phylogenetic information to weight transcriptomic differences can be misleading (Bastide et al., 2023). Herein, we first performed intra‐species transcriptomic analyses and then compared the results across species in search of similarities/differences. This approach has the advantage of avoiding normalization problems across species—particularly when using whole body data—but it has the caveat of accumulating trade‐off errors of the independent analyses, as for each species test, we would randomly expect independent false positive and false negative results. Due to our conservative data analyses pipeline (overlapping two programs for differential analyses, using conserved FDR and FC cut‐offs), we expect our results to be mostly impacted by false negatives. Thus, transcripts differentially regulated between the two conditions may not be detected as so statistically, and consequently, matches of common DET across species will be reduced. Moreover, we have chosen to pool whole‐body samples from multiple individuals and colonies to reduce individual variation biases and to focus on overall changes in expression tendencies. That means that transcripts with reduced expression and/or with tissue‐specific variations were overseen in our results. We also found that the quality of the transcriptome assembly was affected by using the reference genome, thus species whose genomes were available had less fragmented transcriptome assemblies, as suggested by the quality analyses in Table 2. This could affect our results in two ways, first by reducing the gene counts of fragmented transcripts in certain species, thus hindering their identification as differentially expressed, and second by interfering with transcripts annotation. Based on the differences in BUSCO completeness between transcriptomes with and without the genome‐guided assembly, we expect that around 10% of the transcripts could be affected in this way. Still, we have chosen to use the reference genomes to improve some assemblies, first because we had at least one desert and one temperate species with a reference genome, allowing us to account at some level for the impact of different assembly strategies in the overall expression pattern, and secondly, because we wanted to make available to the community the best reference transcriptome sets possible. These caveats could hardly be avoided due to the before mentioned reasons, and overall, they do not hinder the main conclusions of our study.

In summary, our results show that as opposed to temperate ant species, desert ants have reduced gene expression alterations even when under extreme heat stress. Furthermore, they support the hypothesis that desert species evolved convergently two transcriptomic strategies to withstand heat stress: in *C*. *holgerseni* and *Mel*. *Bagoti*, a constitutive level of thermoprotective genes reduces the need for great changes in gene expression when temperature increases; contrastingly, in *C*. *bombycina* and *O*. *robustior*, a larger number of genes are differentially regulated in response to increasing heat. These two molecular responses are associated with the survival pattern of these ants: the species with fewer gene expression alterations have a small variability in individual survival, with all workers dying within a two‐degree interval, while species with greater expression responses experience a survival reduction from 100% to 0% within a larger temperature interval of six degrees. The interplay among body size, energetic metabolism, and heat tolerance in the evolution of these two strategies deserves further exploration. Overall, the heat stress response involved mostly conserved genes and pathways across the studied species, still new genes were co‐expressed during heat stress offering an opportunity for adaptative gene neofunctionalization. Finally, this study shows that, in some circumstances, transcriptional differences between conditions may be insufficient to understand putative adaptative mechanisms if they are not contextualized within a phylogenetic perspective. Indeed, the expression of some genes may be constitutive and, therefore, these genes will not be detected as differentially regulated even though they may be functionally relevant to species adaptation. Clearly, transcriptome studies should consider an evolutionary approach for comparative data analyses including multiple species from different phylogenetical backgrounds.

## AUTHOR CONTRIBUTIONS

**Natalia de Souza Araujo:** Data curation (lead); formal analysis (lead); investigation (equal); methodology (equal); validation (equal); visualization (equal); writing – original draft (equal); writing – review and editing (lead). **Rémy Perez:** Conceptualization (supporting); data curation (supporting); formal analysis (supporting); investigation (equal); methodology (equal); project administration (supporting); validation (equal); visualization (equal); writing – original draft (equal); writing – review and editing (supporting). **Quentin Willot:** Conceptualization (supporting); investigation (supporting); methodology (supporting); writing – original draft (supporting). **Matthieu DeFrance:** Conceptualization (supporting); data curation (supporting); formal analysis (supporting); investigation (supporting); methodology (supporting); project administration (supporting); supervision (supporting); writing – original draft (supporting). **Serge Aaron:** Conceptualization (lead); funding acquisition (lead); methodology (supporting); project administration (lead); supervision (lead); writing – original draft (equal); writing – review and editing (supporting).

## FUNDING INFORMATION

This work was supported by the Belgian *Fonds National pour la Recherche Scientifique* (grant numbers J.0151.16 to SA, 40005980 to NSA and T.0140.18 to RP) and the Universté Libre de Bruxelles (FER 2017 and 2021).

## CONFLICT OF INTEREST STATEMENT

The authors declare that they have no competing interests.

## Supporting information


Appendix S1.

Figure S5.

Figure S14.

Figure S15.

Figure S16.

Figure S17.

Figure S18.

Figure S19.

## Data Availability

Sequenced data and reference transcriptome assemblies for the studied ant species are available at NCBI under the Bioproject PRJNA632584; the annotations for these assemblies and scripts used are accessible at GitHub (https://github.com/nat2bee/Cataglyphis_HS). Supplementary datasets are available at Dryad under the doi: 10.5061/dryad.sn02v6x9h.
