# Supplementary material for: Facing lethal temperatures: Heat‐shock response in desert and temperate ants
Source: Ecol Evol. 2023 Sep 14;13(9):e10438. doi: 10.1002/ece3.10438 (PMC10500329; doi:10.1002/ece3.10438)
Supplement: Supplementary file 1 — Appendix S1. [file ECE3-13-e10438-s001.docx]

**Supporting Information for:**

**Facing lethal temperatures: heat shock response in desert and temperate ants**

Natalia de Souza Araujo^1¶ *^, Rémy Perez^1¶^, Quentin Willot^1,3^, Matthieu DeFrance^2^ and Serge Aron^1^

^1^ Department of Evolutionary Biology & Ecology, Université Libre de Bruxelles. Avenue F.D. Roosevelt, 50. B-1050 Brussels, Belgium

^2^ Interuniversity Institute of Bioinformatics in Brussels, Université Libre de Bruxelles. B-1050 Brussels, Belgium

^3^ Zoophysiology, Department of Biology, Aarhus University, 8000, Aarhus-C, Denmark

^¶^ These authors contributed equally

^*^ Corresponding author

Natalia de Souza Araujo

**e-mail:** [Natalia.De.Souza.Araujo@ulb.be](mailto:Natalia.De.Souza.Araujo@ulb.be)

**This PDF file includes:**

Table S1

Figures S1 and S2

**Other supporting materials for this manuscript include the following:**

Datasets S2 to S21

Supplementary files S2 to S13, and S20 to S22 are deposited on Dryad (<https://doi.org/10.5061/dryad.sn02v6x9h>).

**Table S1.** GO terms enriched among the differentially expressed transcripts under heat stress in each species.

| **Species** | **GO ID** | **Term** | **Annotated** | **Significant** | **Expected** | **Classic Fisher**  **p-value** |
| --- | --- | --- | --- | --- | --- | --- |
| ***C. bombycina*** | GO:1903699 | tarsal gland development | 1 | 1 | 0 | 0.0044 |
|  | GO:0046697 | decidualization | 1 | 1 | 0 | 0.0044 |
|  | GO:1903709 | uterine gland development | 1 | 1 | 0 | 0.0044 |
|  | GO:0002674 | negative regulation of acute inflammatory response | 1 | 1 | 0 | 0.0044 |
|  | GO:0050953 | sensory perception of light stimulus | 100 | 2 | 0.44 | 0.0087 |
|  | GO:0007288 | sperm axoneme assembly | 2 | 1 | 0.01 | 0.0088 |
|  | GO:0060426 | lung vasculature development | 2 | 1 | 0.01 | 0.0088 |
|  | GO:0061038 | uterus morphogenesis | 2 | 1 | 0.01 | 0.0088 |
|  | GO:0042487 | regulation of odontogenesis of dentin-containing tooth | 2 | 1 | 0.01 | 0.0088 |
|  | GO:1903929 | primary palate development | 2 | 1 | 0.01 | 0.0088 |
|  | GO:0001886 | endothelial cell morphogenesis | 2 | 1 | 0.01 | 0.0088 |
|  | GO:0043568 | positive regulation of insulin-like growth factor receptor signaling pathway | 2 | 1 | 0.01 | 0.0088 |
|  | GO:0036334 | epidermal stem cell homeostasis | 2 | 1 | 0.01 | 0.0088 |
|  | GO:0034405 | response to fluid shear stress | 2 | 1 | 0.01 | 0.0088 |
| ***C. holgerseni*** | GO:0006879 | cellular iron ion homeostasis | 15 | 2 | 0.03 | 0.00035 |
|  | GO:0006826 | iron ion transport | 18 | 2 | 0.03 | 0.00051 |
|  | GO:0042773 | ATP synthesis coupled electron transport | 15 | 2 | 0.03 | 0.00535 |
|  | GO:0051638 | barbed-end actin filament uncapping | 4 | 1 | 0.01 | 0.00762 |
|  | GO:0050830 | defense response to Gram-positive bacterium | 4 | 1 | 0.01 | 0.00762 |
|  | GO:2000813 | negative regulation of barbed-end actin filament capping | 4 | 1 | 0.01 | 0.00762 |
|  | GO:0006122 | mitochondrial electron transport, ubiquinol to cytochrome c | 5 | 1 | 0.01 | 0.00951 |
| ***Mel. bagoti*** | GO:0022904 | respiratory electron transport chain | 35 | 4 | 0.12 | 0.00046 |
|  | GO:1904749 | regulation of protein localization to nucleolus | 2 | 1 | 0.01 | 0.00658 |
| ***O. robustior*** | GO:0031047 | gene silencing by RNA | 72 | 7 | 1.21 | 5.5e-06 |
|  | GO:0042026 | protein refolding | 9 | 4 | 0.15 | 9.0e-06 |
|  | GO:0034605 | cellular response to heat | 23 | 5 | 0.39 | 3.3e-05 |
|  | GO:0006457 | protein folding | 89 | 11 | 1.5 | 0.00024 |
|  | GO:0006275 | regulation of DNA replication | 20 | 4 | 0.34 | 0.00030 |
|  | GO:0051085 | chaperone cofactor-dependent protein refolding | 4 | 2 | 0.07 | 0.00165 |
|  | GO:0006265 | DNA topological change | 18 | 3 | 0.3 | 0.00317 |
|  | GO:0071923 | negative regulation of cohesin loading | 7 | 2 | 0.12 | 0.00560 |
|  | GO:0051312 | chromosome decondensation | 7 | 2 | 0.12 | 0.00560 |
| ***F. fusca*** | GO:0007169 | transmembrane receptor protein tyrosine kinase signaling pathway | 211 | 38 | 16.4 | 2.2e-12 |
|  | GO:0042026 | protein refolding | 18 | 12 | 1.4 | 5.4e-10 |
|  | GO:0034605 | cellular response to heat | 32 | 14 | 2.49 | 5.0e-08 |
|  | GO:0006458 | 'de novo' protein folding | 14 | 11 | 1.09 | 2.7e-06 |
|  | GO:0006457 | protein folding | 109 | 33 | 8.47 | 3.1e-06 |
|  | GO:0051085 | chaperone cofactor-dependent protein refolding | 8 | 6 | 0.62 | 5.3e-06 |
|  | GO:0045041 | protein import into mitochondrial intermembrane space | 6 | 5 | 0.47 | 1.6e-05 |
|  | GO:0050808 | synapse organization | 299 | 26 | 23.24 | 7.2e-05 |
|  | GO:0015074 | DNA integration | 178 | 29 | 13.84 | 0.00010 |
|  | GO:0050829 | defense response to Gram-negative bacterium | 34 | 10 | 2.64 | 0.00018 |
|  | GO:1902037 | negative regulation of hematopoietic stem cell differentiation | 3 | 3 | 0.23 | 0.00047 |
|  | GO:0010529 | negative regulation of transposition | 6 | 4 | 0.47 | 0.00048 |
|  | GO:0008637 | apoptotic mitochondrial changes | 37 | 11 | 2.88 | 0.00050 |
|  | GO:0090074 | negative regulation of protein homodimerization activity | 7 | 4 | 0.54 | 0.00105 |
|  | GO:0031547 | brain-derived neurotrophic factor receptor signaling pathway | 12 | 5 | 0.93 | 0.00140 |
|  | GO:2000481 | positive regulation of cAMP-dependent protein kinase activity | 12 | 5 | 0.93 | 0.00140 |
|  | GO:0048022 | negative regulation of melanin biosynthetic process | 12 | 5 | 0.93 | 0.00140 |
|  | GO:2001224 | positive regulation of neuron migration | 12 | 5 | 0.93 | 0.00140 |
|  | GO:2001214 | positive regulation of vasculogenesis | 12 | 5 | 0.93 | 0.00140 |
|  | GO:2000670 | positive regulation of dendritic cell apoptotic process | 12 | 5 | 0.93 | 0.00140 |
|  | GO:0016246 | RNA interference | 22 | 7 | 1.71 | 0.00195 |
|  | GO:0008050 | female courtship behavior | 8 | 4 | 0.62 | 0.00197 |
|  | GO:1901888 | regulation of cell junction assembly | 153 | 17 | 11.89 | 0.00211 |
|  | GO:0043950 | positive regulation of cAMP-mediated signaling | 13 | 5 | 1.01 | 0.00213 |
|  | GO:0021884 | forebrain neuron development | 26 | 6 | 2.02 | 0.00310 |
|  | GO:0071880 | adenylate cyclase-activating adrenergic receptor signaling pathway | 14 | 5 | 1.09 | 0.00310 |
|  | GO:0030950 | establishment or maintenance of actin cytoskeleton polarity | 9 | 4 | 0.7 | 0.00332 |
|  | GO:0051146 | striated muscle cell differentiation | 166 | 11 | 12.9 | 0.00341 |
|  | GO:0045089 | positive regulation of innate immune response | 46 | 12 | 3.58 | 0.00370 |
|  | GO:0050774 | negative regulation of dendrite morphogenesis | 27 | 7 | 2.1 | 0.00373 |
|  | GO:0046475 | glycerophospholipid catabolic process | 5 | 3 | 0.39 | 0.00415 |
|  | GO:0032486 | Rap protein signal transduction | 15 | 5 | 1.17 | 0.00435 |
|  | GO:0032092 | positive regulation of protein binding | 28 | 7 | 2.18 | 0.00465 |
|  | GO:0048036 | central complex development | 10 | 4 | 0.78 | 0.00520 |
|  | GO:0007257 | activation of JUN kinase activity | 16 | 5 | 1.24 | 0.00593 |
|  | GO:0038180 | nerve growth factor signaling pathway | 16 | 5 | 1.24 | 0.00593 |
|  | GO:0070868 | obsolete heterochromatin organization involved in chromatin silencing | 16 | 5 | 1.24 | 0.00593 |
|  | GO:0016042 | lipid catabolic process | 134 | 16 | 10.42 | 0.00596 |
|  | GO:0006336 | DNA replication-independent chromatin assembly | 11 | 4 | 0.86 | 0.00768 |
|  | GO:0097345 | mitochondrial outer membrane permeabilization | 10 | 4 | 0.78 | 0.00781 |
|  | GO:0042247 | estestablishment of planar polarity of follicular epithelium | 6 | 3 | 0.47 | 0.00783 |
|  | GO:0015771 | trehalose transport | 6 | 3 | 0.47 | 0.00783 |
|  | GO:1902463 | protein localization to cell leading edge | 6 | 3 | 0.47 | 0.00783 |
|  | GO:0030033 | microvillus assembly | 17 | 5 | 1.32 | 0.00788 |
|  | GO:0044331 | cell-cell adhesion mediated by cadherin | 31 | 7 | 2.41 | 0.00842 |
|  | GO:0051491 | positive regulation of filopodium assembly | 47 | 9 | 3.65 | 0.00923 |
| ***Myr. sabuleti*** | GO:0042026 | protein refolding | 18 | 11 | 2.08 | 6.9e-07 |
|  | GO:0006458 | 'de novo' protein folding | 22 | 11 | 2.54 | 3.7e-06 |
|  | GO:1990481 | mRNA pseudouridine synthesis | 5 | 5 | 0.58 | 2.0e-05 |
|  | GO:0000495 | box H/ACA snoRNA 3'-end processing | 5 | 5 | 0.58 | 2.0e-05 |
|  | GO:0031120 | snRNA pseudouridine synthesis | 5 | 5 | 0.58 | 2.0e-05 |
|  | GO:0031118 | rRNA pseudouridine synthesis | 5 | 5 | 0.58 | 2.0e-05 |
|  | GO:0045041 | protein import into mitochondrial intermembrane space | 13 | 8 | 1.5 | 2.3e-05 |
|  | GO:0034605 | cellular response to heat | 31 | 12 | 3.58 | 9.2e-05 |
|  | GO:0008637 | apoptotic mitochondrial changes | 37 | 11 | 4.28 | 0.00011 |
|  | GO:0035220 | wing disc development | 130 | 23 | 15.02 | 0.00046 |
|  | GO:0006000 | fructose metabolic process | 5 | 4 | 0.58 | 0.00081 |
|  | GO:0006003 | fructose 2,6-bisphosphate metabolic process | 5 | 4 | 0.58 | 0.00081 |
|  | GO:0007614 | short-term memory | 29 | 10 | 3.35 | 0.00103 |
|  | GO:0016567 | protein ubiquitination | 221 | 30 | 25.54 | 0.00105 |
|  | GO:0007281 | germ cell development | 304 | 39 | 35.13 | 0.00117 |
|  | GO:0044351 | macropinocytosis | 3 | 3 | 0.35 | 0.00154 |
|  | GO:0040040 | thermosensory behavior | 13 | 6 | 1.5 | 0.00196 |
|  | GO:0006610 | ribosomal protein import into nucleus | 19 | 7 | 2.2 | 0.00387 |
|  | GO:0019941 | modification-dependent protein catabolic process | 341 | 43 | 39.41 | 0.00464 |
|  | GO:0046084 | adenine biosynthetic process | 7 | 4 | 0.81 | 0.00465 |
|  | GO:0031291 | Ran protein signal transduction | 7 | 4 | 0.81 | 0.00465 |
|  | GO:0001654 | eye development | 185 | 21 | 21.38 | 0.00472 |
|  | GO:0010886 | positive regulation of cholesterol storage | 4 | 3 | 0.46 | 0.00563 |
|  | GO:0034383 | low-density lipoprotein particle clearance | 4 | 3 | 0.46 | 0.00563 |
|  | GO:0030953 | astral microtubule organization | 4 | 3 | 0.46 | 0.00563 |
|  | GO:0043097 | pyrimidine nucleoside salvage | 4 | 3 | 0.46 | 0.00563 |
|  | GO:1901741 | positive regulation of myoblast fusion | 4 | 3 | 0.46 | 0.00563 |
|  | GO:0061512 | protein localization to cilium | 16 | 6 | 1.85 | 0.00675 |
|  | GO:0007619 | courtship behavior | 58 | 15 | 6.7 | 0.00711 |
|  | GO:0046958 | nonassociative learning | 24 | 7 | 2.77 | 0.00842 |
|  | GO:0006465 | signal peptide processing | 8 | 4 | 0.92 | 0.00846 |
|  | GO:0006198 | cAMP catabolic process | 8 | 4 | 0.92 | 0.00846 |
|  | GO:0010738 | regulation of protein kinase A signaling | 8 | 4 | 0.92 | 0.00846 |

**Fig S1.** Median lethal temperature (LT50) after 3 hours of exposure for all species during the heat-stress treatment. LT50_3h and 95% confidence intervals were calculated using a simple logistic regression of death probability. A ratio test was used for pairwise comparisons between species. All species display significantly different values (*p* < 0.05).


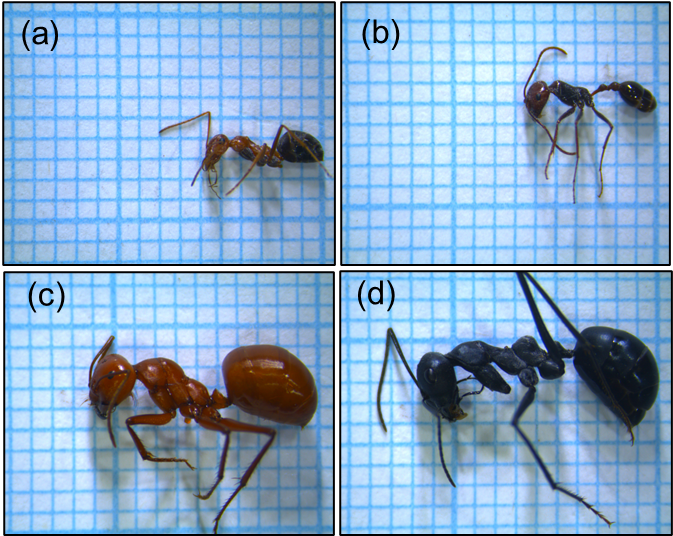


**Figure S2.** Workers from the desert ant species studied (a) *Cataglyphis bombycina*, (b) *Ocymyrmex robustior* (c) *Melophorus bagoti*, and (d) *Cataglyphis holgerseni*. The body mass of the pictured workers was respectively: (a) 5.2 mg, (b) 4.6 mg (c) 59.8 mg, and (d) 52.8 mg. The workers shown are representative of most foragers we observed.
