# Supplementary figures and images for: Facing lethal temperatures: Heat‐shock response in desert and temperate ants
Source: Ecol Evol. 2023 Sep 14;13(9):e10438. doi: 10.1002/ece3.10438 (PMC10500329; doi:10.1002/ece3.10438)

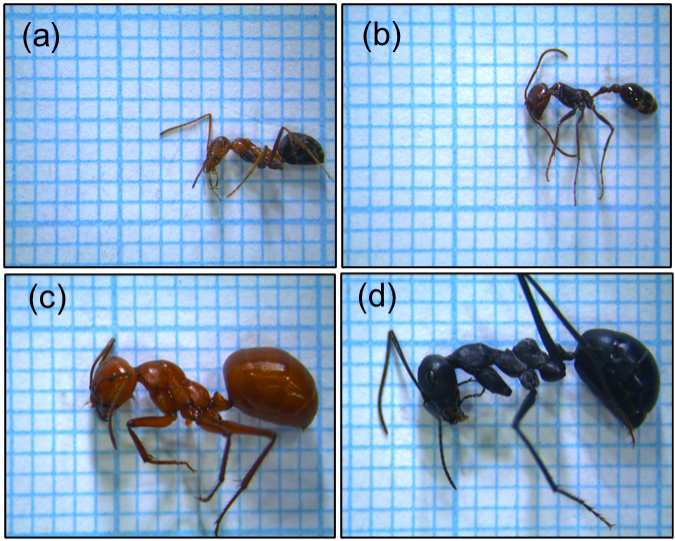

Supplement: Supplementary file 2 — Figure S5. [file ECE3-13-e10438-s008.png]

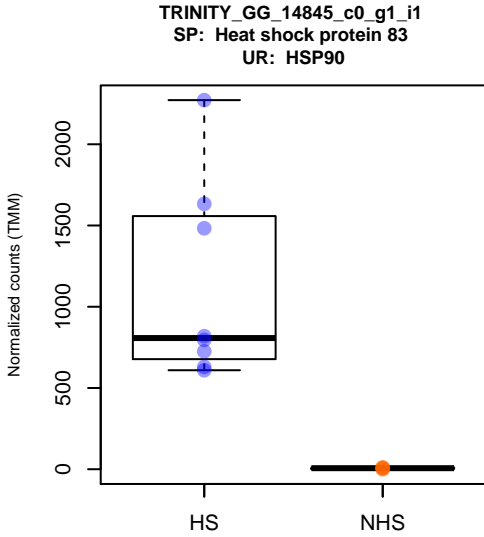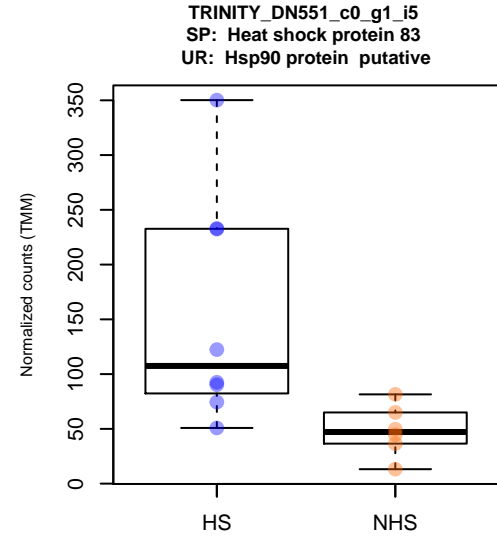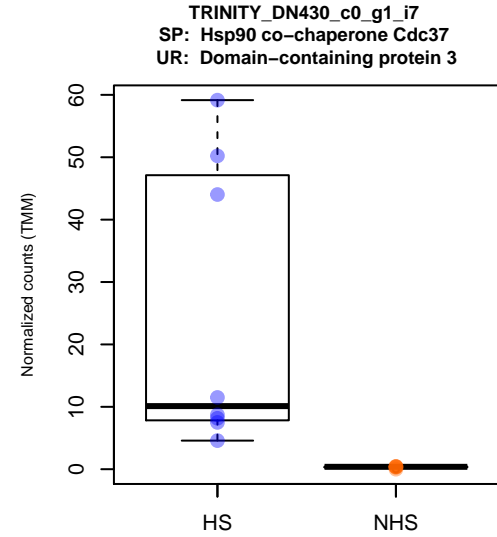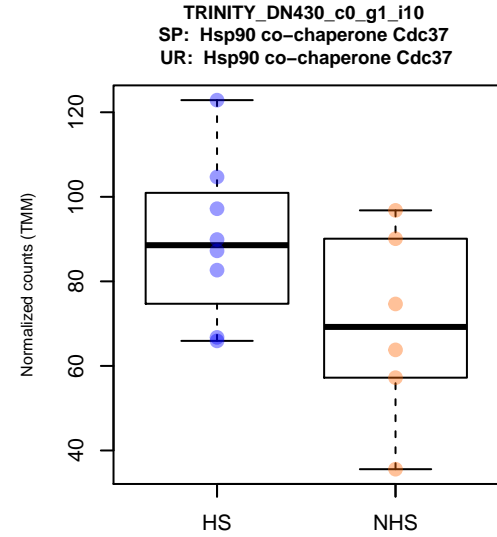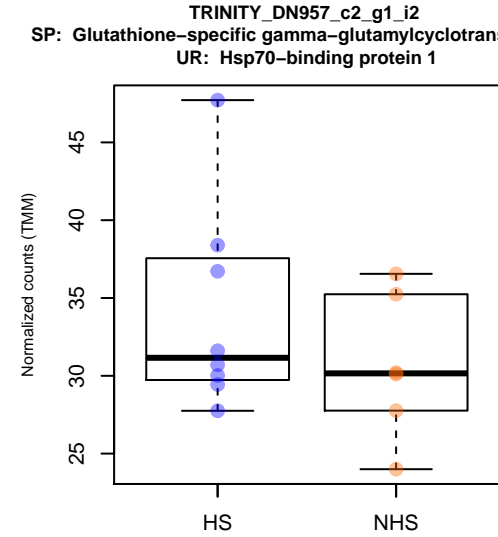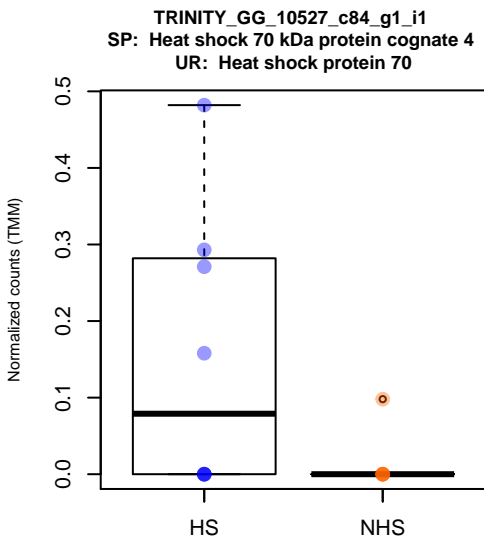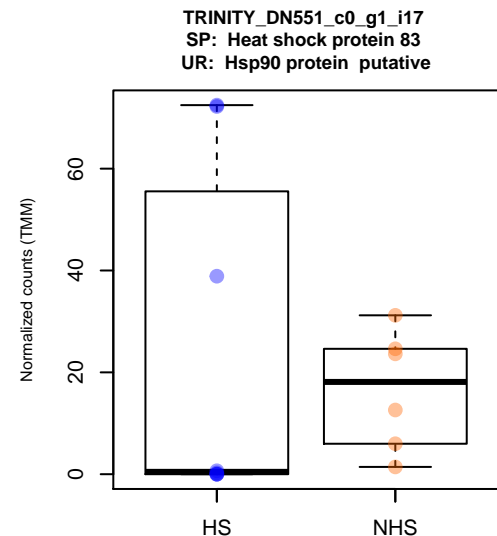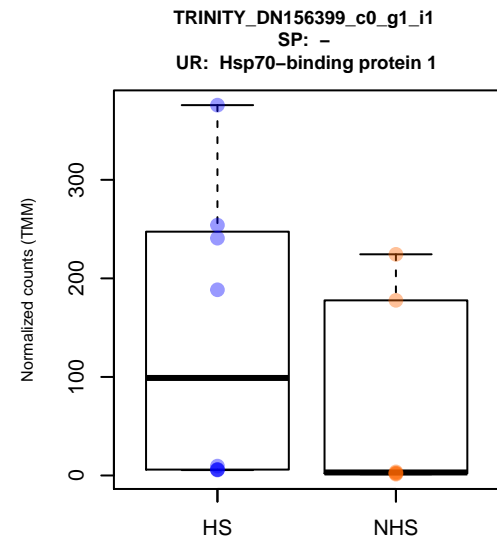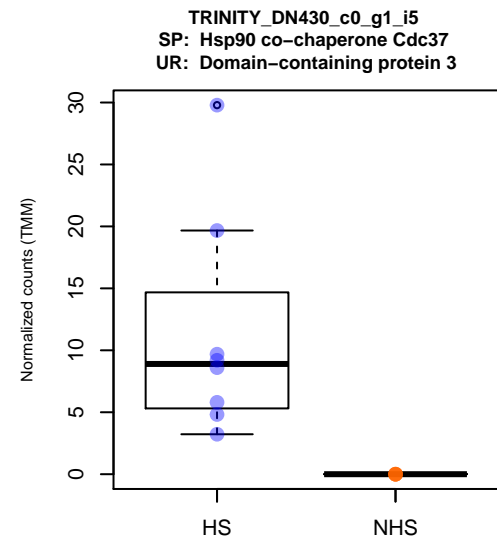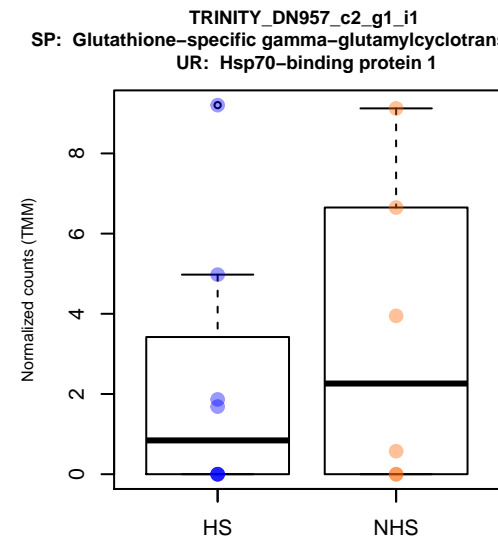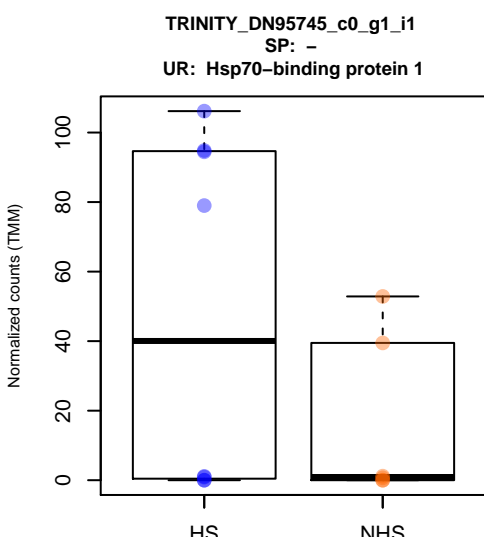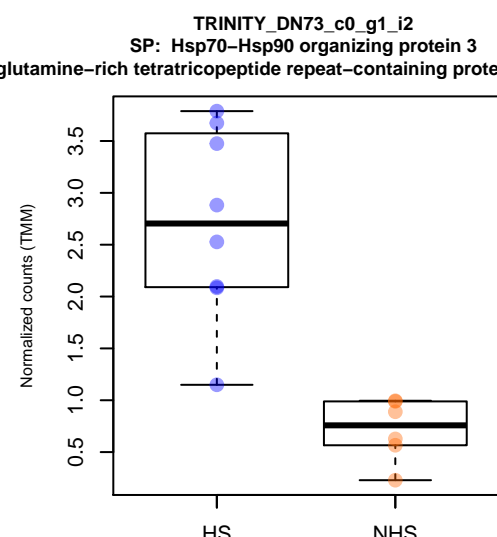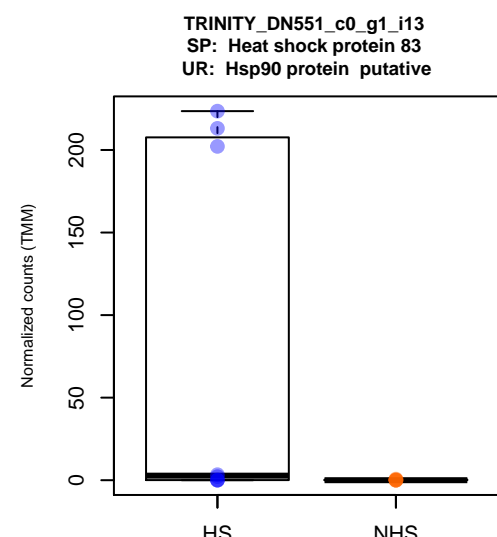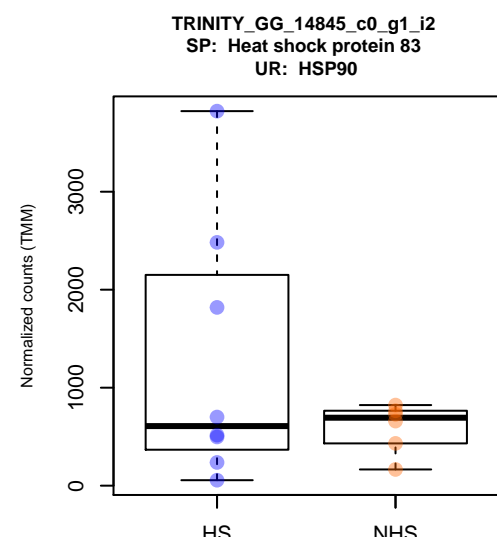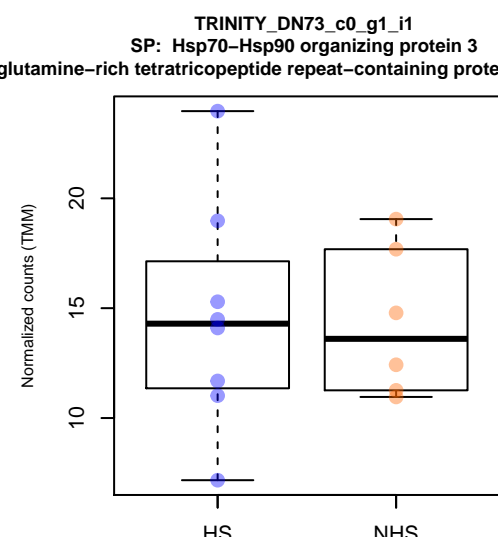

Supplement: Supplementary file 3 — Figure S14. [file ECE3-13-e10438-s003.pdf]

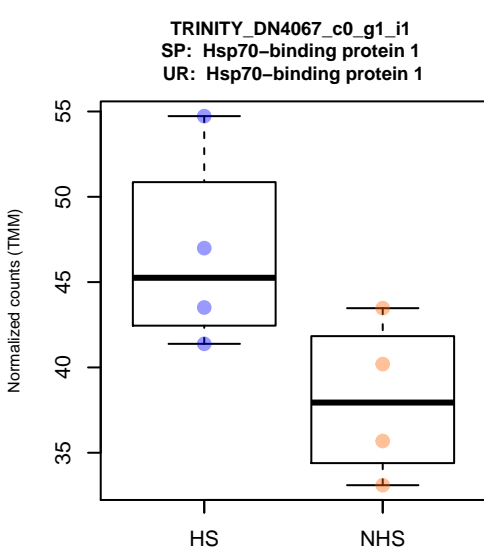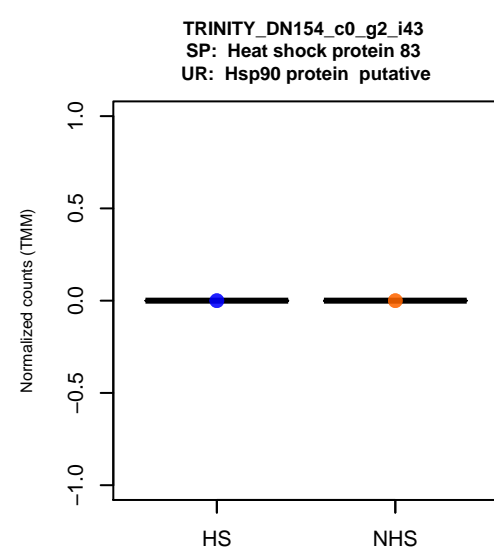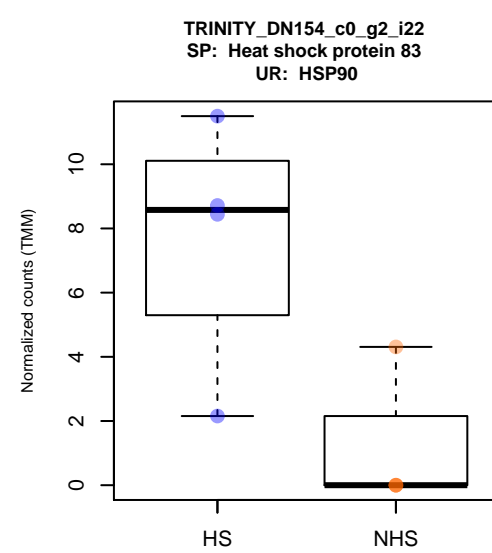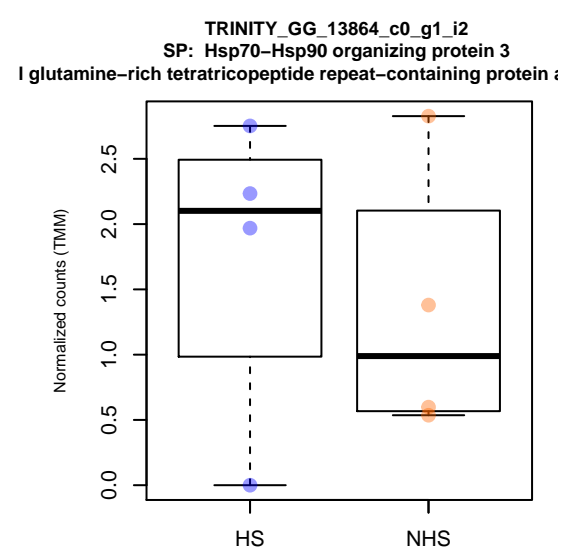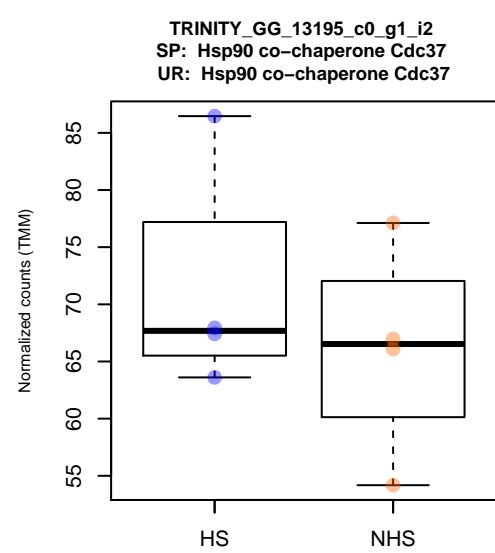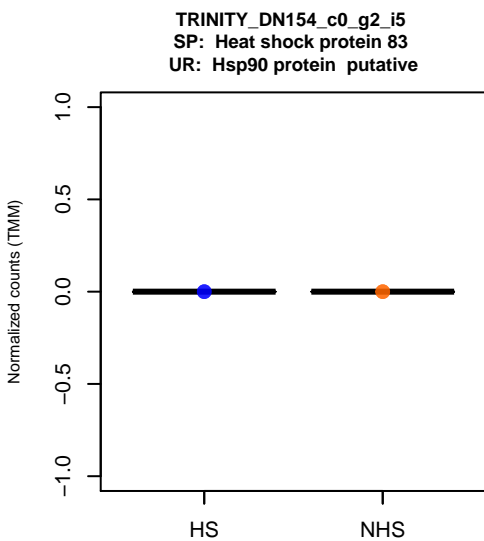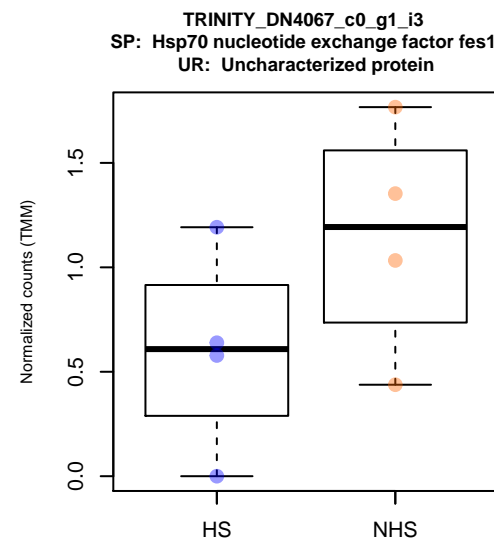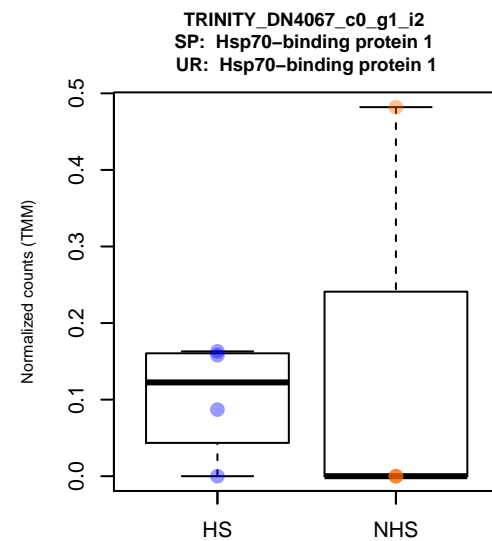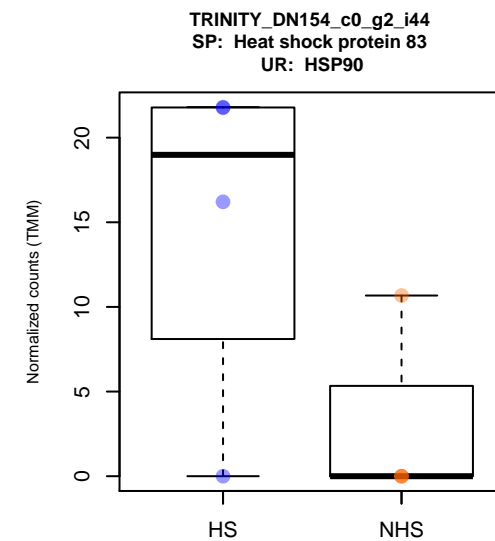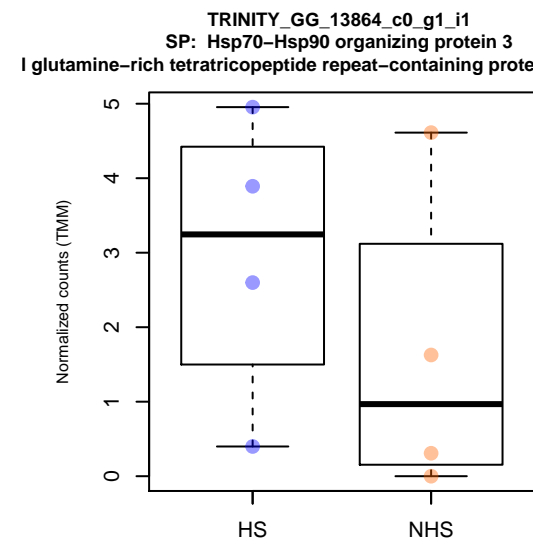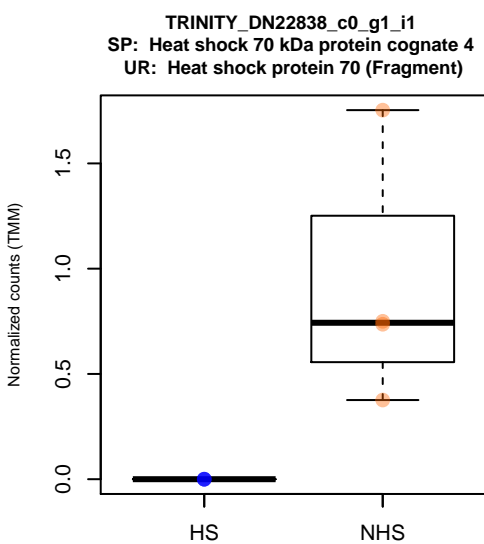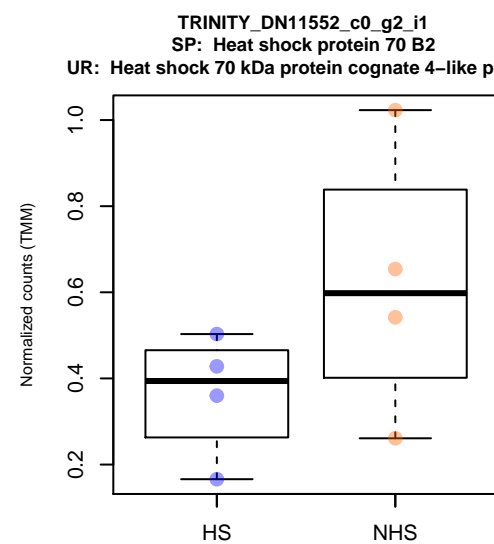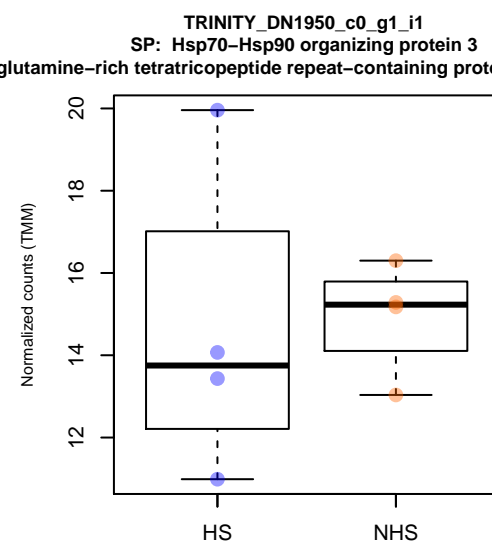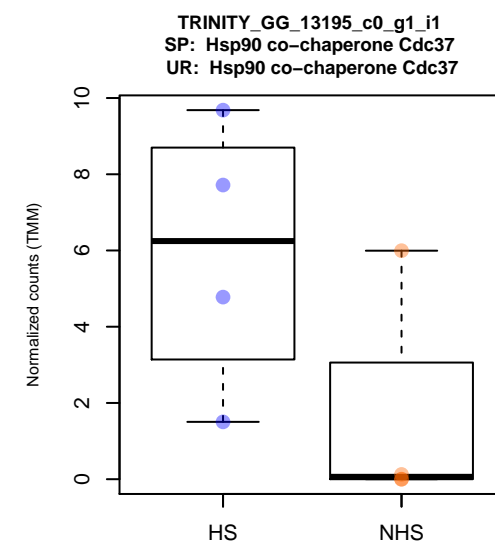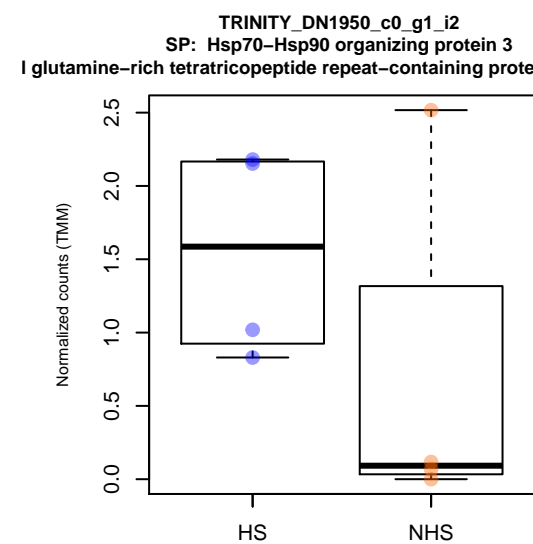

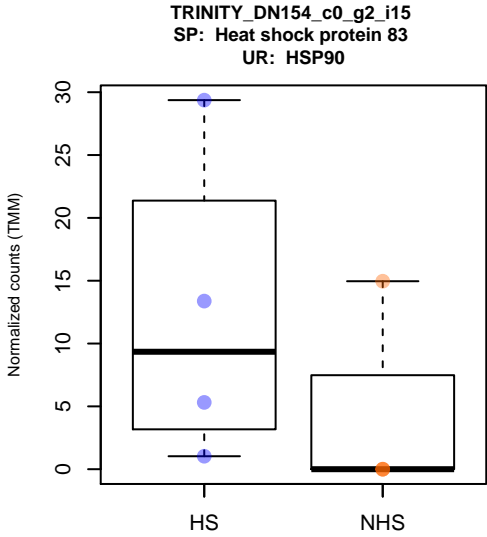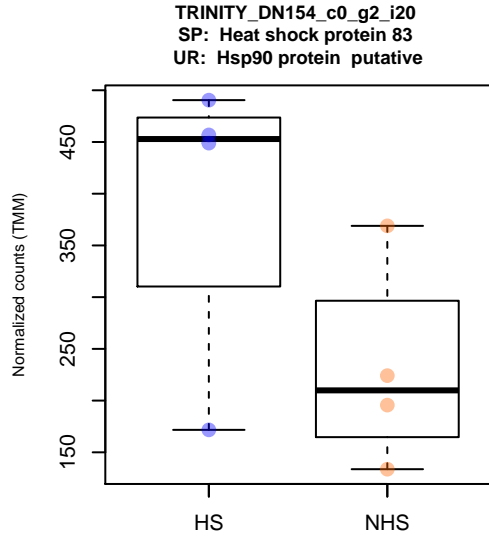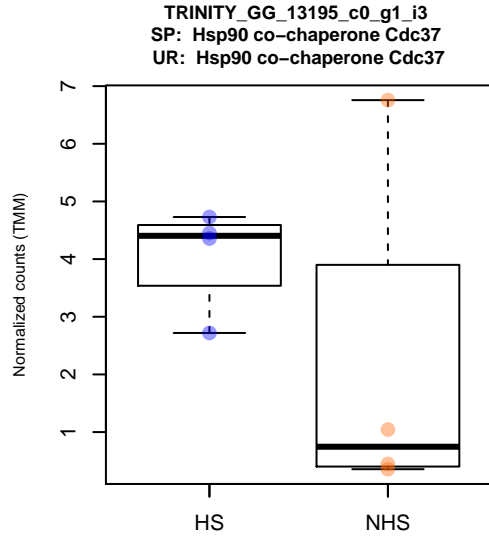

Supplement: Supplementary file 4 — Figure S15. [file ECE3-13-e10438-s007.pdf]

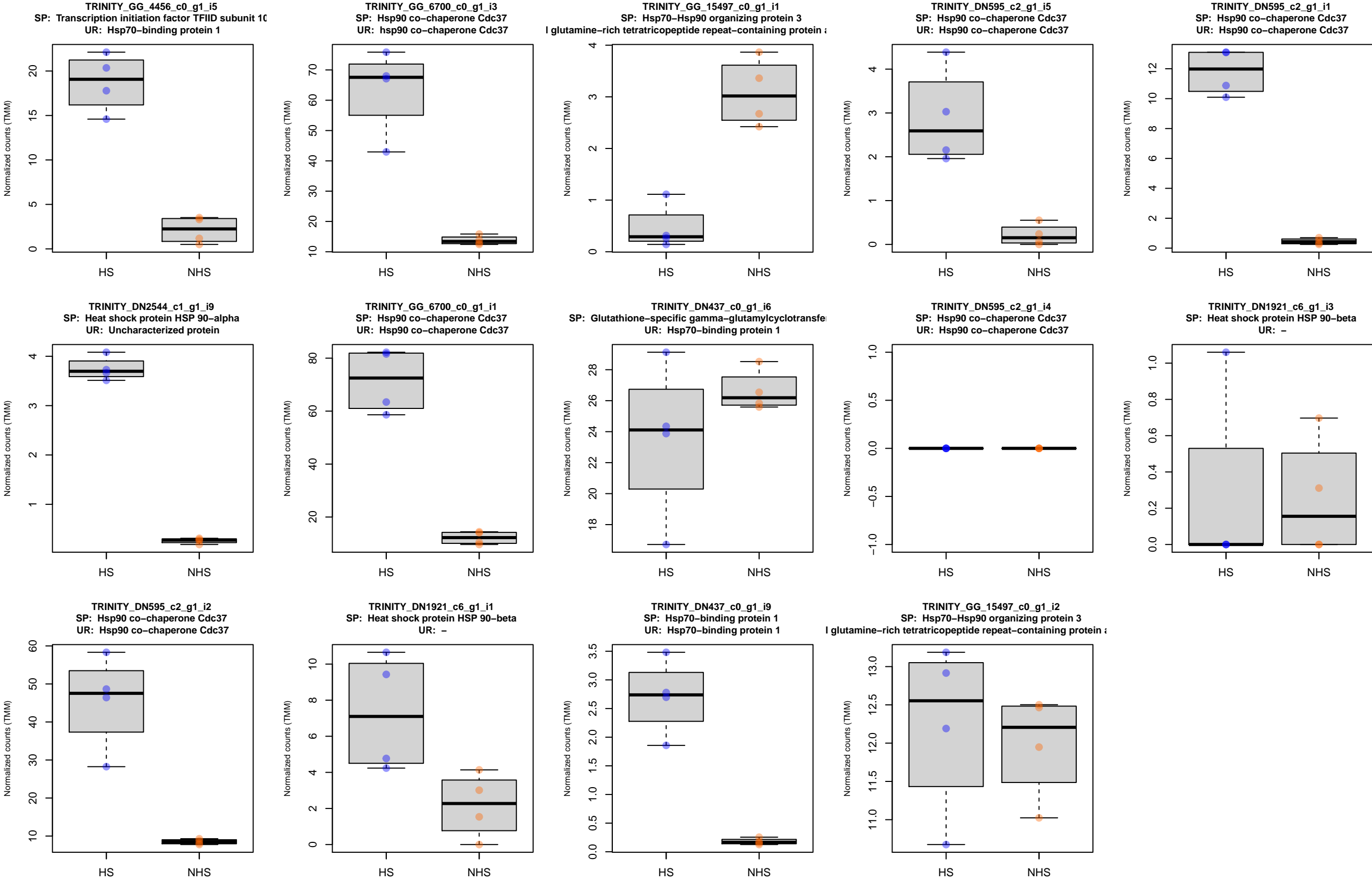

Supplement: Supplementary file 5 — Figure S16. [file ECE3-13-e10438-s006.pdf]

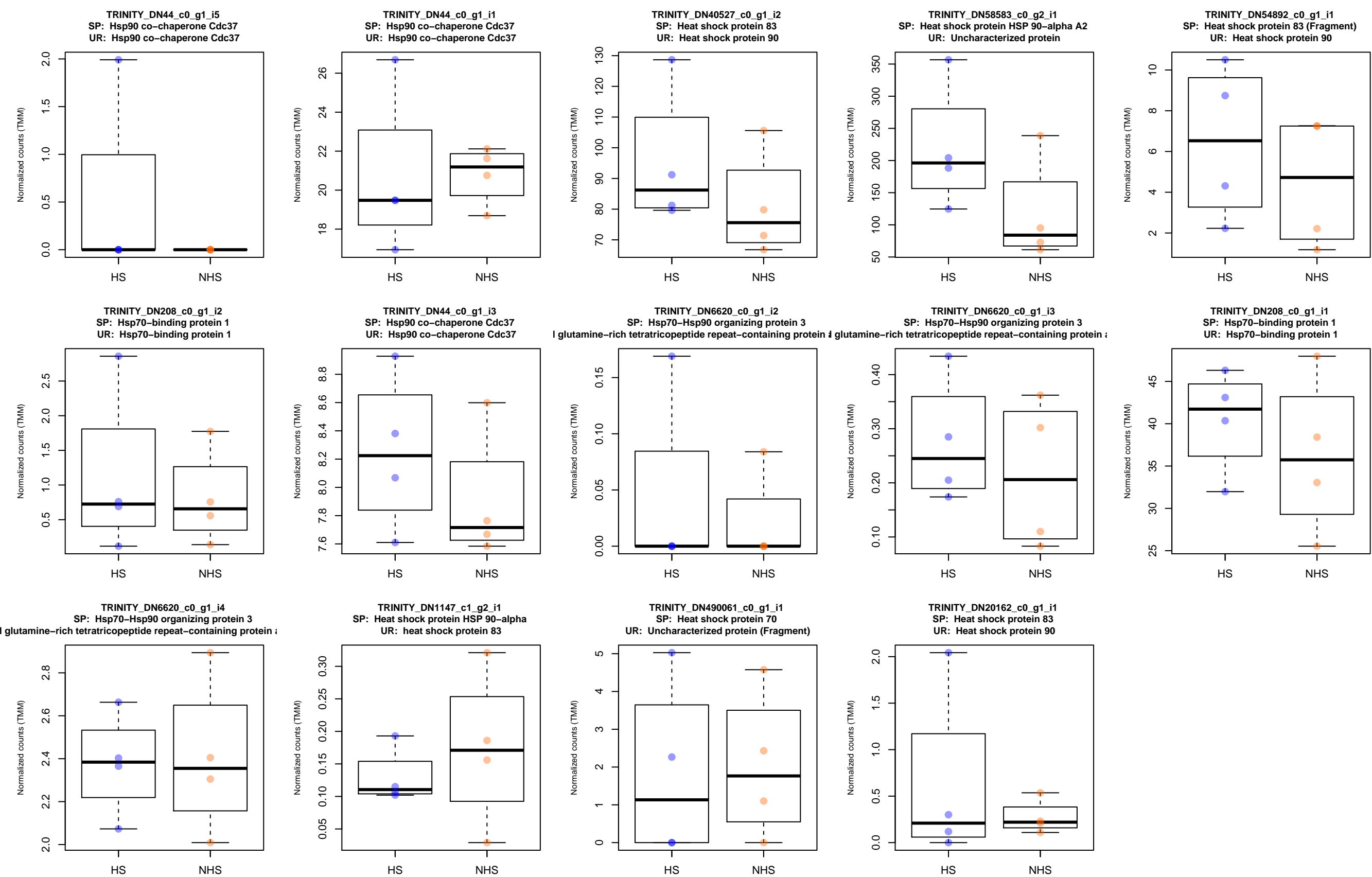

Supplement: Supplementary file 6 — Figure S17. [file ECE3-13-e10438-s004.pdf]

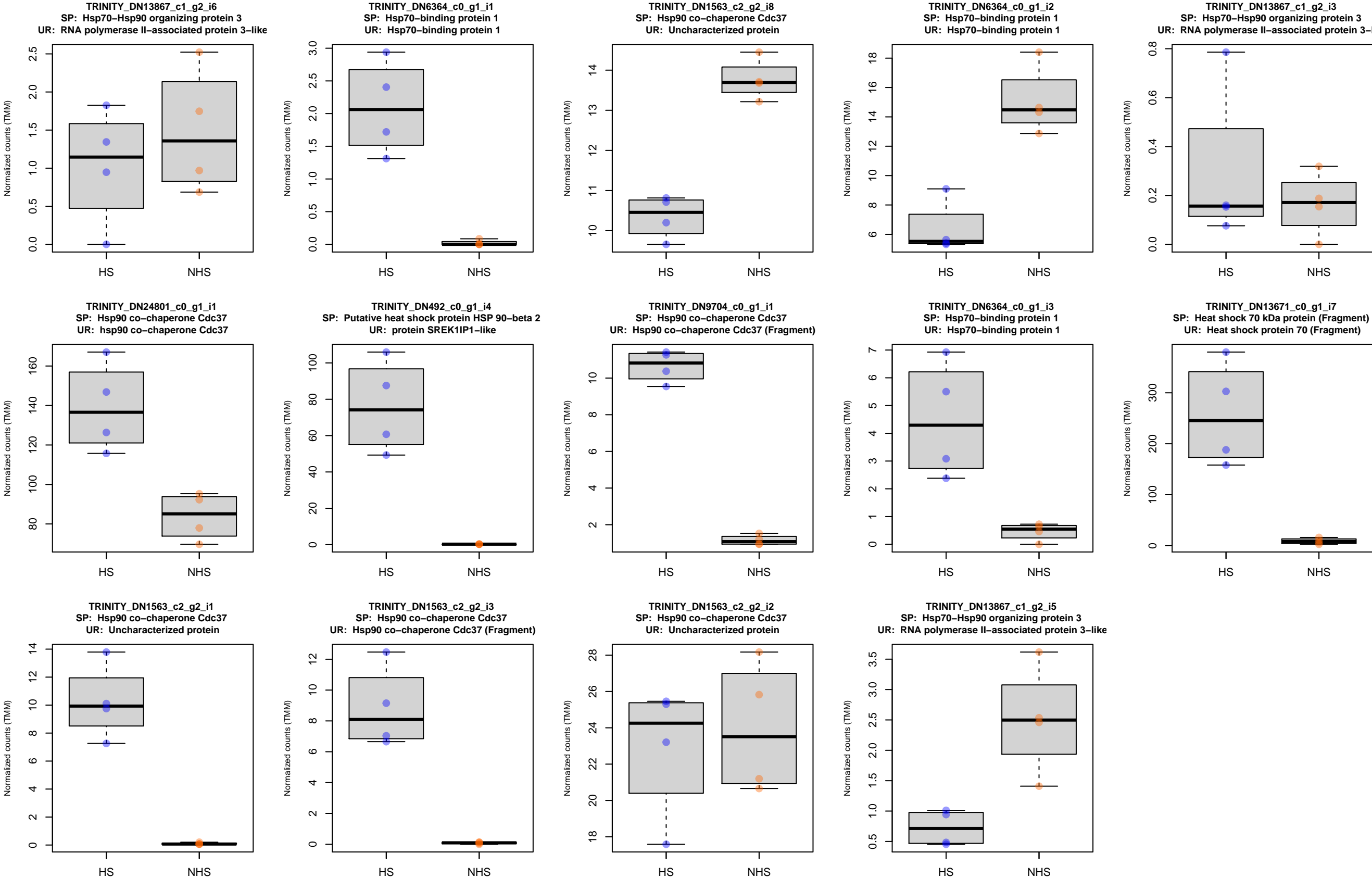

Supplement: Supplementary file 7 — Figure S18. [file ECE3-13-e10438-s002.pdf]

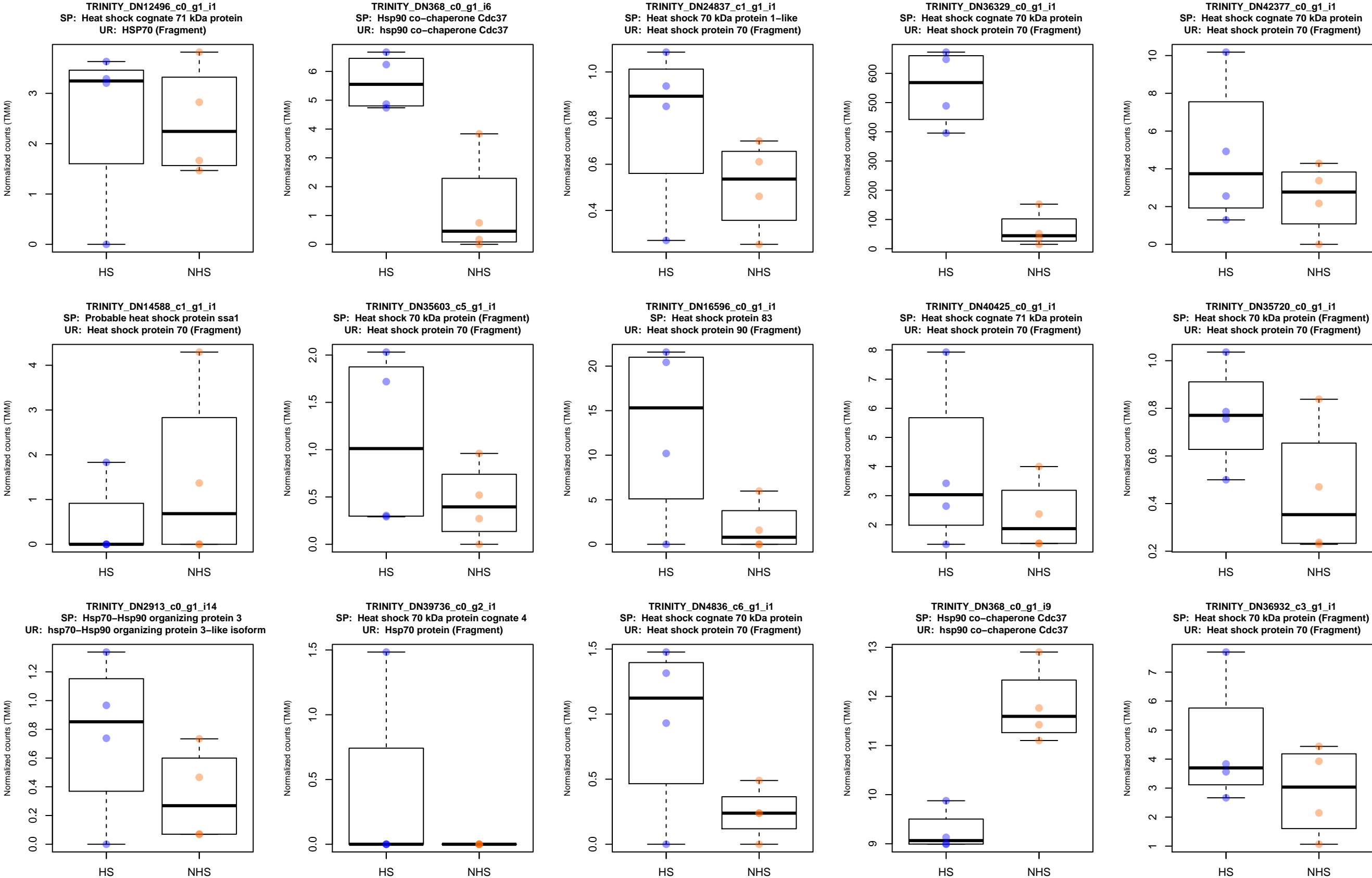

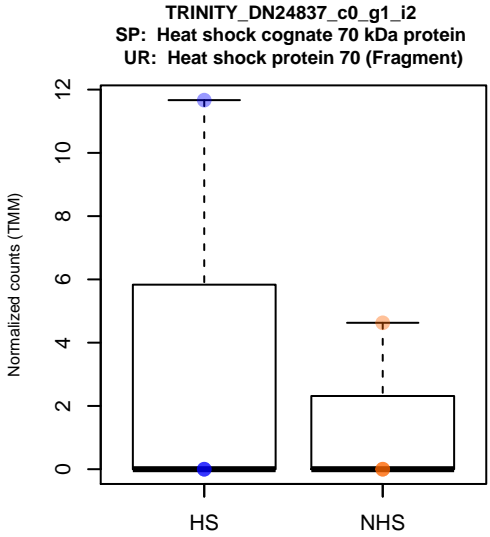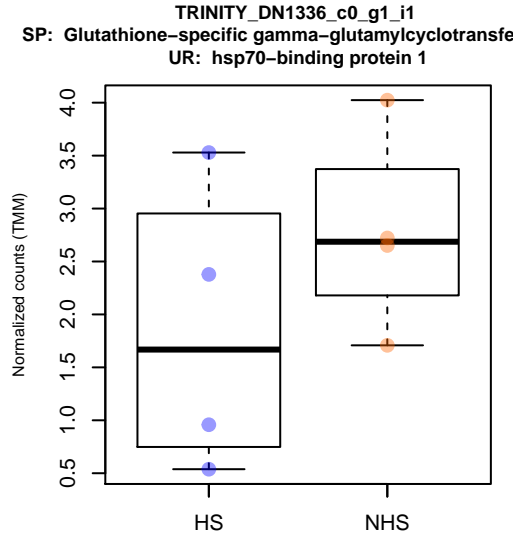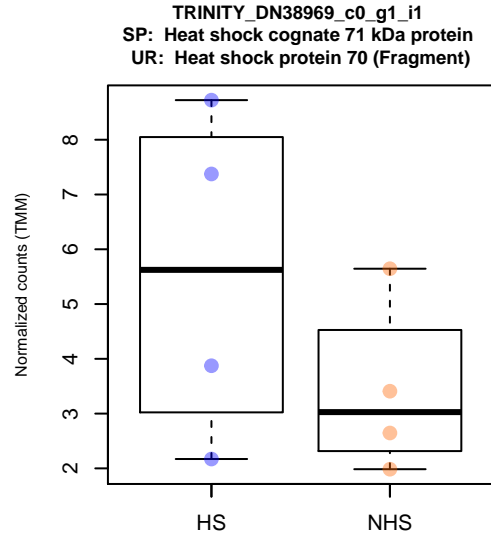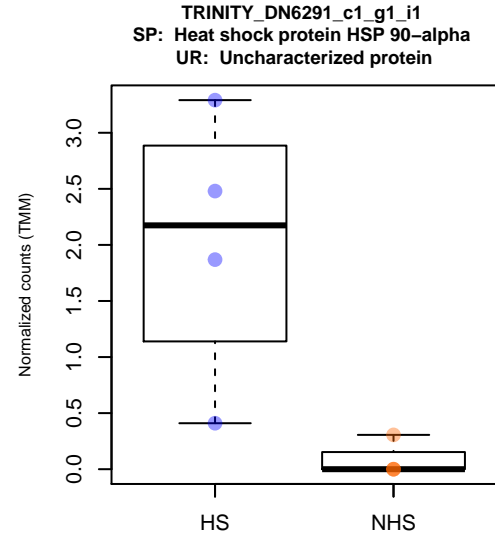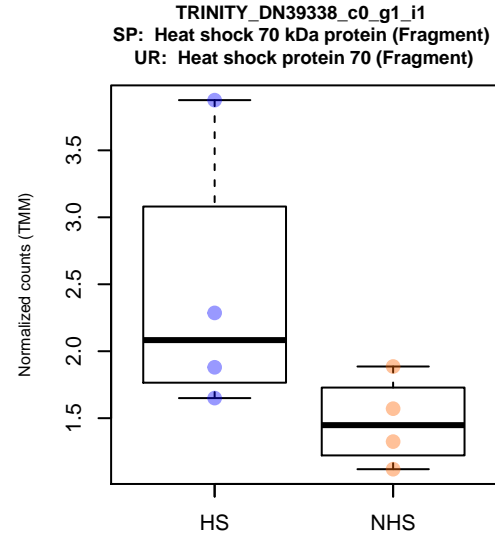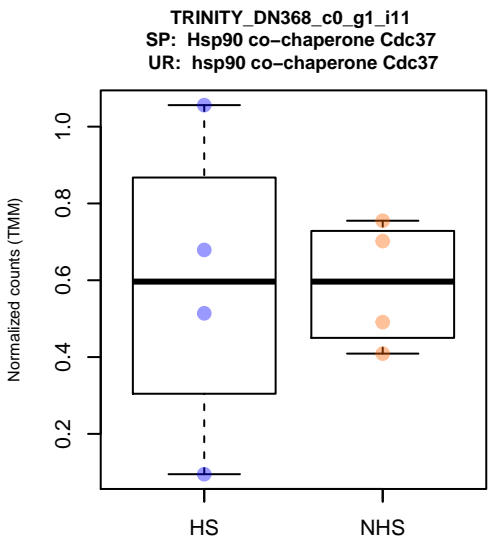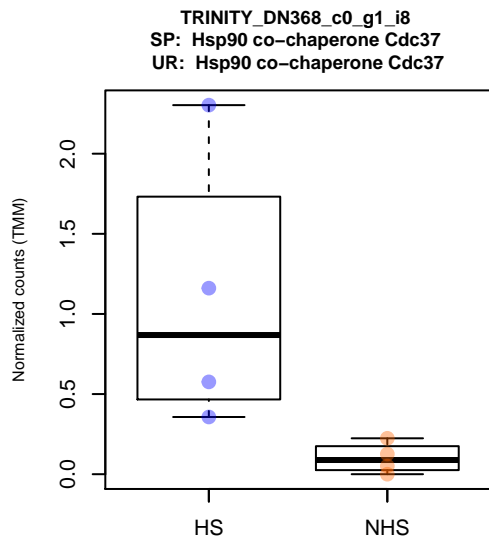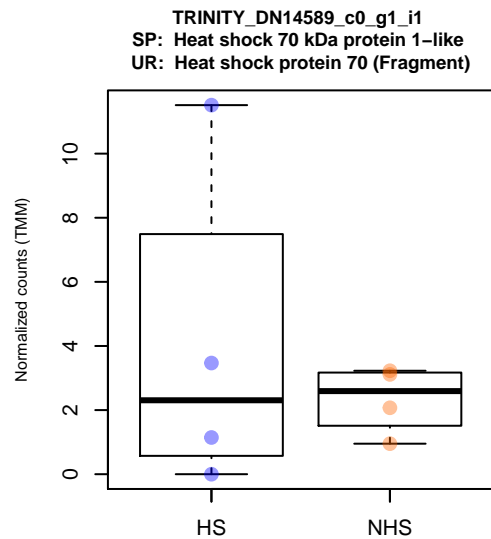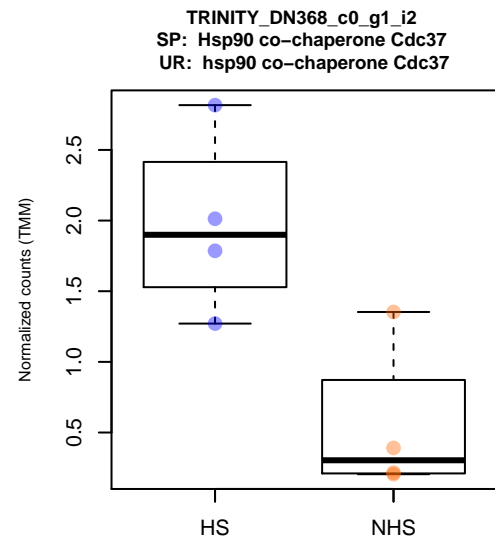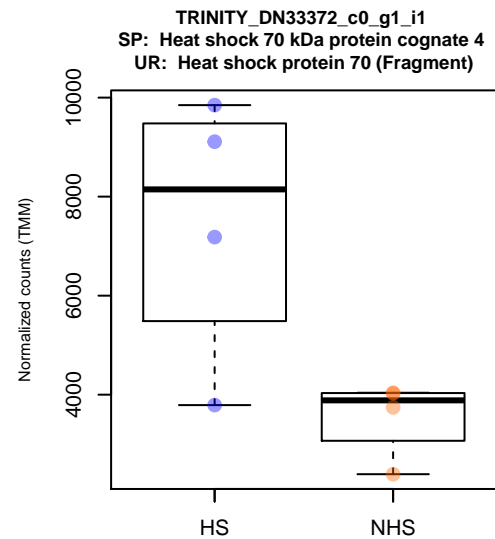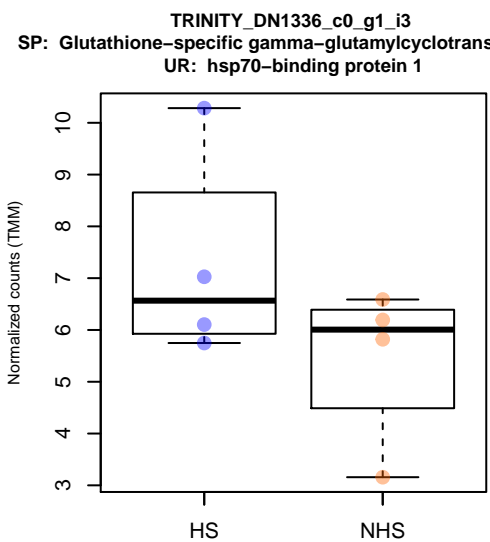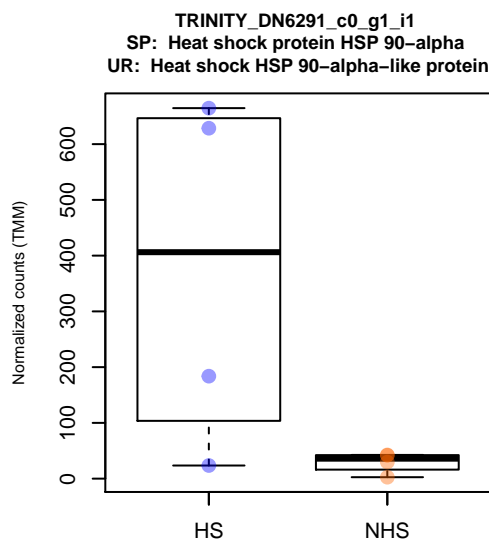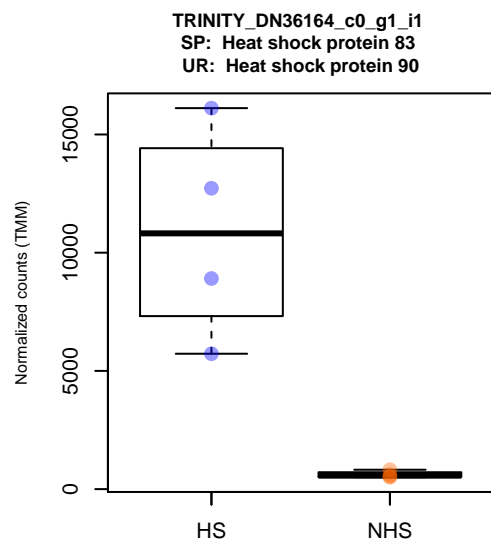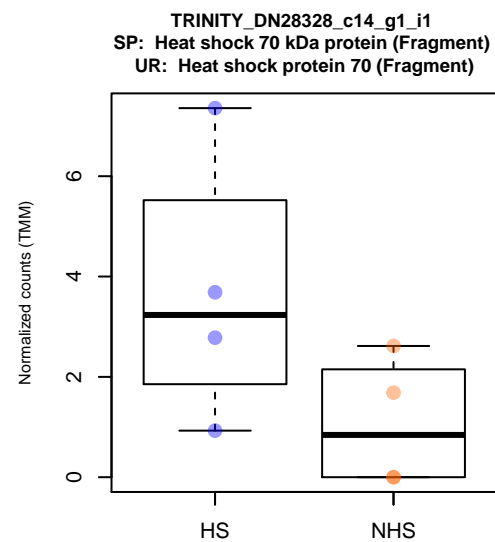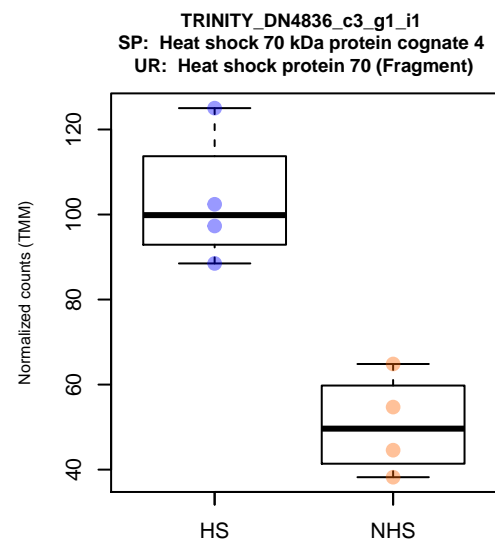

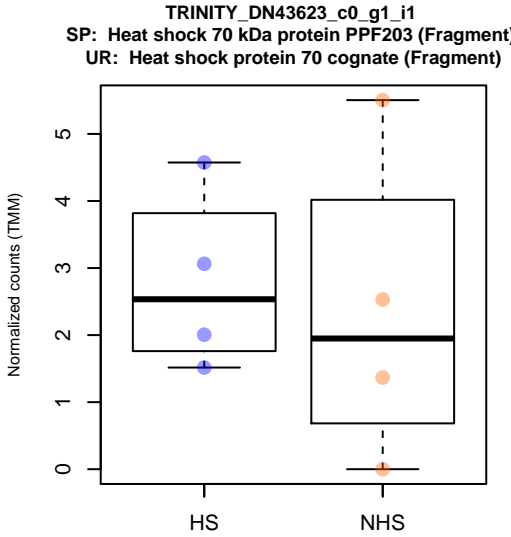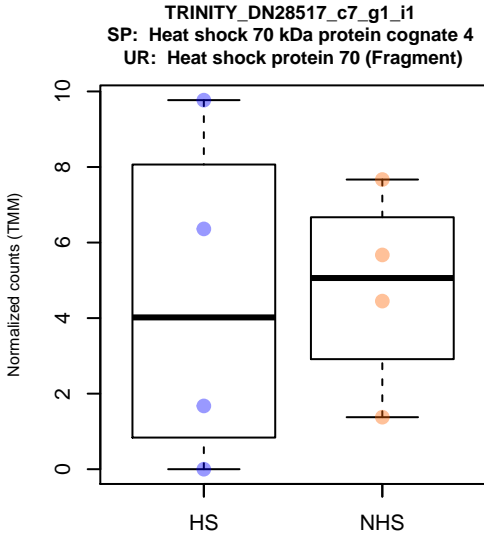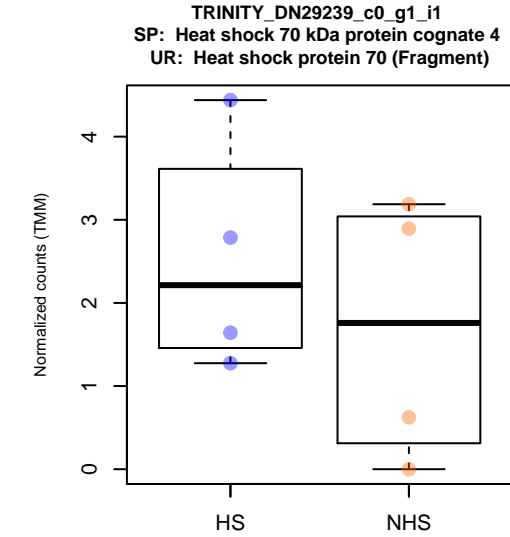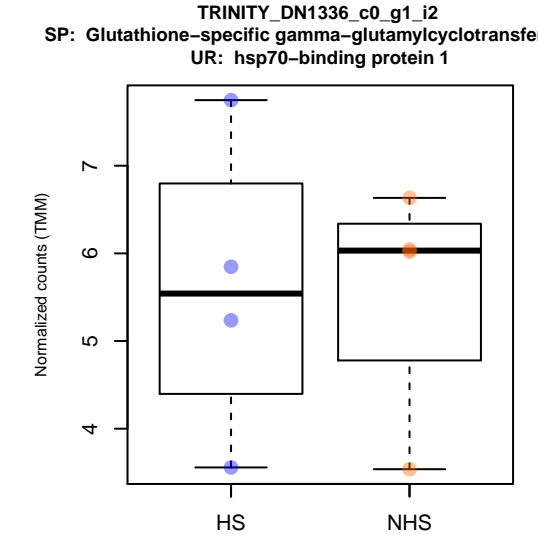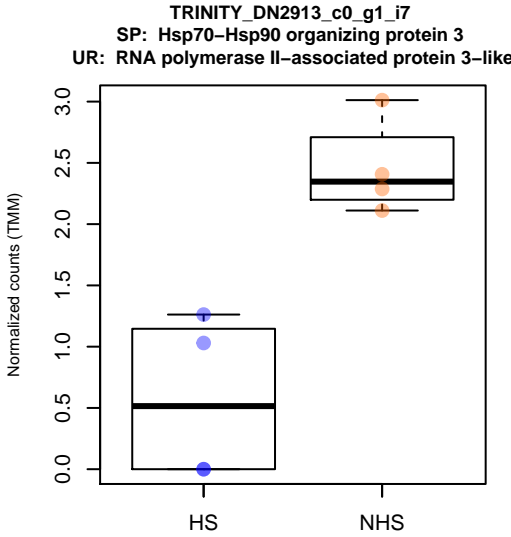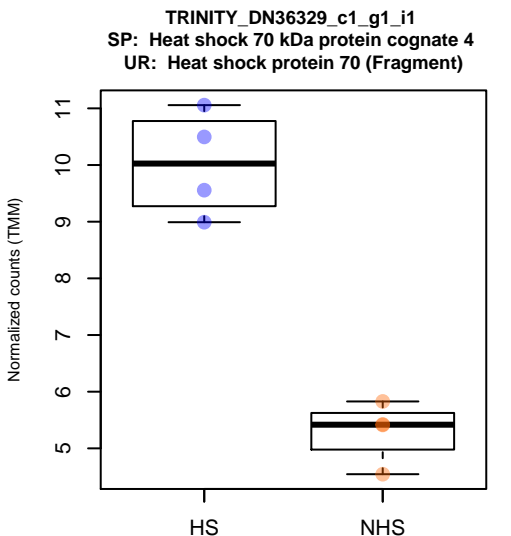

Supplement: Supplementary file 8 — Figure S19. [file ECE3-13-e10438-s005.pdf]
